# MtDNA copy number enrichment is associated with poor prognosis and eosinophilic morphology in clear cell renal cell carcinoma

**DOI:** 10.3389/pore.2025.1612172

**Published:** 2025-07-23

**Authors:** Sarah Bellal, Cyrielle Rolley, Jeremy Richard, Nolwenn Bounaix, Vincent Le Corre, Marie-Christine Copin, Odile Blanchet, Pierre Bigot, Vincent Procaccio, Céline Bris

**Affiliations:** ^1^ Department of Pathology, Angers University Hospital, Angers, France; ^2^ Univ Angers, INSERM U1083, CNRS UMR6015, MITOVASC, SFR ICAT, Angers, France; ^3^ Department of Urology, UroCCR, Angers University Hospital, Angers, France; ^4^ Biological Resource Centre, BB-0033-00038, Angers University Hospital, Angers, France; ^5^ Genetics Department, Angers University Hospital, Angers, France

**Keywords:** clear cell renal cell carcinoma, mtDNA copy number, prognosis, 786-O cell line, ethidium bromide, eosinophilic clear cell renal cell carcinoma, mitochondrial genome

## Abstract

Clear cell renal cell carcinoma (ccRCC) is the most common renal malignancy. However, the combined clinical and biological scores commonly used to predict the clinical outcome are imperfect and need improvement. The main goal of our study was to assess the effect of mtDNA genetics on the prognosis of ccRCC patients and to explore morphologic correlation. Mitochondrial DNA copy number (mtDNAcn) variation between tumor and paired matched healthy kidney tissue was assessed by real-time quantitative PCR and expressed as a ratio in 105 patients. According to this median ratio, the cohort was divided into two groups: “LOW” (n = 53) and “HIGH” (n = 52). Cancer-Specific Survival (CSS) and Disease-Free Survival were assessed in each group. The tumor samples were classified into two subtypes (Clear or Eosinophilic cells) according to the cytoplasmic morphology. CSS was significantly reduced in the “HIGH” than in the “LOW” group with respective 5-year survival rates: 78.7% (CI 95: 64.8–95.5) and 95.5% (CI 95 87.1–100.0) (Hazard Ratio: 7.4 (CI 95: 1.9–29.9, p = 0.027*) in multivariate analysis, including pathological classification, tumor size, International Society of Urological Pathology grade, lymphovascular invasion, dedifferentiated pattern, necrosis and adjuvant therapy. Next-generation sequencing of mtDNA was performed on 14 tumors and matched healthy kidney tissue. No hotspot mutation or redundant large deletion was found. None of the variants or large deletions identified had an impact on prognosis. MtDNAcn variation in tumor relative to normal kidney appears as an independent prognostic factor in ccRCC, which was also associated with eosinophilic morphology. MtDNA content could be considered an additional prognostic factor, in combination with other predictive parameters. Furthermore, these results underline the importance of the role of mitochondria in ccRCC and the need for further functional studies to understand the pathophysiological mechanisms better and consider therapies targeting mitochondrial metabolism.

## Introduction

Clear cell renal cell carcinoma (ccRCC) is the most common renal cancer type (90%), with nearly 300,000 (2.6%) new cases yearly and higher incidence in developed countries [1, 2]. CcRCC is heterogeneous and characterized by clear or eosinophilic cells arranged in trabecular cords or nest architecture in a delicate stroma richly vascularized. The risk of recurrence post-nephrectomy for locally advanced kidney cancer is notably variable, with key factors including T stage, ISUP (International Society of Urological Pathology) grade, and the presence of necrosis or symptoms at diagnosis [3–5]. Various prognostic nomograms, such as UISS (University of California Los Angeles Integrated Staging System), ASSURE, and Leibovitch, have incorporated these clinical and pathological data [3, 6–10]. It is noteworthy that, as of now, no biological markers are employed in clinical practice to augment the prognostic precision of existing models.

As metabolic reprogramming is one of the hallmarks of cancer and mitochondria play a central role in metabolism, their role in carcinogenesis has been evaluated for years [11, 12]. CcRCC is characterized by a metabolic shift from mitochondrial oxidation to anaerobic glycolysis in the presence of oxygen, known as the Warburg effect [13–15], driven by the loss of function of the Von Hippel-Lindau gene *(VHL)* and the Hypoxia-inducible Factor (HIF) stabilization [16, 17].

In ccRCC, somatic variants in the nuclear genome accumulate according to the phylogenetic clone model, retaining a driver strain defect: the bi-allelic inactivation of *VHL* [18]. Other mutated genes are numerous and most often affect polybromo-1 (*PBRM1*), SET domain containing 2 (*SETD2*) or BRCA1-associated protein 1 (*BAP1*) genes [18–20], which are involved in methylation or chromatin compaction phenomena. Mitochondrial DNA (mtDNA) is a 16,569 bp circular double-stranded DNA molecule, maternally inherited. It encodes 13 polypeptides involved in oxidative phosphorylation (OXPHOS), and regulates reactive oxygen species (ROS) [21]. The accumulation of qualitative alterations and/or quantitative variation of mtDNA has been found to play a crucial role in carcinogenesis and metastasis, among other elements, by modulating cellular metabolism [11, 12]. Variations in mtDNA and their consequences on survival and disease progression are variable among cancers. In cancer subtypes of renal origin (chromophobe carcinoma) or non-renal origin (adrenocortical carcinoma or glioblastoma), high mtDNA copy number (mtDNAcn) is associated with better survival [22–24]. Conversely, a significant increase in mtDNAcn is associated with poorer prognosis in melanoma or breast carcinoma [22, 25]. In ccRCC, the impact of mtDNAcn on prognosis is conflicting. Some studies have shown that an increase in mtDNAcn relative to normal matched normal tissue is associated with a worse prognosis [22]. Still, other studies have suggested that a decrease in mtDNAcn compared to normal paired tissue and mitochondrial respiratory chain activity *in vitro* is associated with increased tumor growth, invasion capacity, and drug resistance mechanisms [26].

The first aim of this study was to assess the influence of mtDNA genetics (a.k.a mitochondrial copy number variation, deletions, and mutations) on the prognosis in patients with ccRCC and to explore histologic correlation. As an exploratory second aim, we looked at the impact of mtDNA depletion on cell proliferation in a ccRCC cell line.

## Materials and methods

### Population of the study

We retrospectively included 144 patients treated at Angers University Hospital from 2011 to 2019 for locally advanced ccRCC. Patients were registered in the UroCCR database (French Research Network for Kidney Cancer, NCT03294563, with the French data protection authority (CNIL) agreement DR-2013-206. Patients’ biological samples were stored in a biocollection of the Biological Resource Center (DC-2014-2224) in Angers Hospital. Written informed consent was obtained from all individuals with the approval of the research ethics committee of Angers University Hospital (authorisation number 2021-014). CcRCC diagnosis was established by an experienced pathologist. Metastatic patients at diagnosis, patients with other histological subtypes, patients who received chemotherapy, immunotherapy or targeted therapy before surgery, or who had less than 2 years of clinical follow-up were excluded. After partial or radical nephrectomy, fresh tumors and matched adjacent-healthy kidney tissue were snap frozen and long-term stored in liquid nitrogen (−196°C) at the biological resource centre (BRC) of Angers Hospital. One piece of the frozen tumoral sample was formalin-fixed and paraffin-embedded (FFPE) as a morphological control.

Disease-free survival (DFS) and Cancer-Specific Survival (CSS) were assessed using the date of recurrence, death, or the last clinical follow-up, respectively. Eight patients who did not die from ccRCC were excluded from CSS analysis. All clinical, pathological, and demographic data used for statistical analyses are available in Supplementary Table S1.

### DNA extraction, mtDNA quantification and mtDNA sequencing

DNA extraction was performed using Qiamp DNA Mini kit (Qiagen, Hilden, Germany) according to manufacturer’s instructions and quantified by Nanodrop^®^2000 (Thermo Fischer Scientific, Waltham, Massachusetts, USA).

Mitochondrial DNA copy number (mtDNAcn) was assessed as previously described [27] by real time quantitative Polymerase Chain Reaction (Q-PCR). For each sample (tumor and healthy kidney tissues), the mtDNAcn was determined by the ratio of the mean copy number of two mitochondrial genes (*MT-CO1* and *MT-ND4*) and two nuclear genes (*B2M*: *beta2-microglobulin and GAPDH: glyceraldehyde 3-phosphate dehydrogenase*). The real-time quantitative PCR (Q-PCR) was performed using the Chromo4 System (Biorad^®^, Hercules, CA, USA) in a 20 μL reaction volume containing ×1 IQ SYBR Green Supermix (Biorad^®^) and a final concentration of 0.5 μM of each gene-specific primer and 3 μL of template. The details of the primers are available in Supplementary Table S2.

For each patient, mtDNAcn variation between the tumor and adjacent normal tissue was expressed as a ratio (Tumor/Healthy Kidney: T/HK). Patients were then subdivided into two groups according to this ratio median: LOW mtDNAcn ratio (≤median) and HIGH mtDNAcn ratio (>median).

High-throughput mtDNA Next Generation Sequencing (NGS) was performed using Ion Torrent Proton, and the signal processing and base calling were done by our in-house bioinformatic pipeline on 14 tumor-healthy tissue pairs as previously described [27]. All somatic mutations in coding or non-coding mtDNA sequences were collected, and variants were classified as homoplasmic (>95% mtDNA) or heteroplasmic (<95%) according to their variant allele frequency. Large mtDNA rearrangements were searched using eKLIPse software [28].

### Morphological and immunochemical analysis

FFPE morphological control was assessed blindly to genetic data. Cytoplasmic eosinophilia was rated microscopically as follows: 0: <5% eosinophilia, 1: 5%–20%, 3+: >90% and 2+: neither 1+ nor 3+ (Supplementary Figure S1). Scores 0 and 1+ were combined into the Clear Cells (CC) group, and scores 2+ and 3+ were encompassed in the Eosinophilic (EO) group. Because of intra-tumoral heterogeneity, ISUP corrected (ISUPc) grade [29] was assessed and assigned to each frozen sample (Supplementary Table S1).

Immunohistochemistry was performed on whole slide sections using an automated immunochemistry system (Leica Bond III, Wetzlar, Germany) with TOMM20 antibody (Translocase of the outer mitochondrial membrane complex subunit 20, Abcam, Cambridge, UK, ab186735, clone EPR15581-54, 1:1000). TOMM20 is an outer mitochondrial membrane protein. Its expression would support that eosinophilic morphology is related to mitochondria rather than other organelles.

### MtDNA depletion in 786-O cell line and cell imaging

786-O CcRCC cell line (ATCC number CRL-1932, Boulogne Billancourt, France) was gradually devoided of mtDNA content using Ethidium Bromide (BET) (Sigma-Aldrich Saint Louis, Missouri, USA) at 200 ng/mL for 7 days. 786-O cells were maintained in Dulbecco’s modified Eagle’s medium (DMEM) with 4.5 g/L glucose (Pan Biotech, Bernolsheim, France), supplemented with 10% of Fœtal Bovine Serum (FBS) (Good Pan, Pan Biotech), 1% of glutamine (Dominique Dutscher, Bernolsheim, France), 1% of uridine (Pan Biotech), 1% of pyruvate (ThermoFisher Scientific) and incubated at 37.5°C under a 5% CO_2_ atmosphere. The culture medium was changed every day.

To standardize the cell culture conditions, 786-O-WT wild type (not depleted) and 786-O-D (mtDNA depleted) cells were cultured under the same supplied medium conditions. Briefly, cells were plated in quadruplets in one plate with three biological replicates. One well was trypsinized (Pan Biotech) and extracted on day 4 to assess the level of mtDNAcn as described above. All remaining wells were trypsinized and extracted on day eight. Cell proliferation was evaluated using live cell imaging (IncuCyte ZOOM system, Sartorius, Gottingen, Germany), taking images every 2 h, and estimated as the ratio between the well’s confluence (%) calculated during 8 days (192 h) reported to the cellular confluence at day 0 by using the Basic Analyzer segmentation mask of the IncuCyte ZOOM software 2015A.

### Statistical analyses

Quantitative data were expressed with median or mean and interquartile range (median or mean; [IQR]). Qualitative data are given as percentages. Survival data are provided with the hazard ratio (HR) and 95% confidence interval (CI95%). Chi-2 test (qualitative data), Mann-Whitney test (quantitative data), log-rank test, and Kaplan-Meier curve (survival data) were performed on SAS JMP 10 software (SAS Institute Inc, NC, USA) or GraphPad Prism 9 (San Diego, USA). When needed, Benjamini and Hochberg’s corrections were performed. P value < 0.05 was considered significant. Multivariate analysis was performed using the Cox model.

## Results

### Constitution of the cohort

**FIGURE 1:**
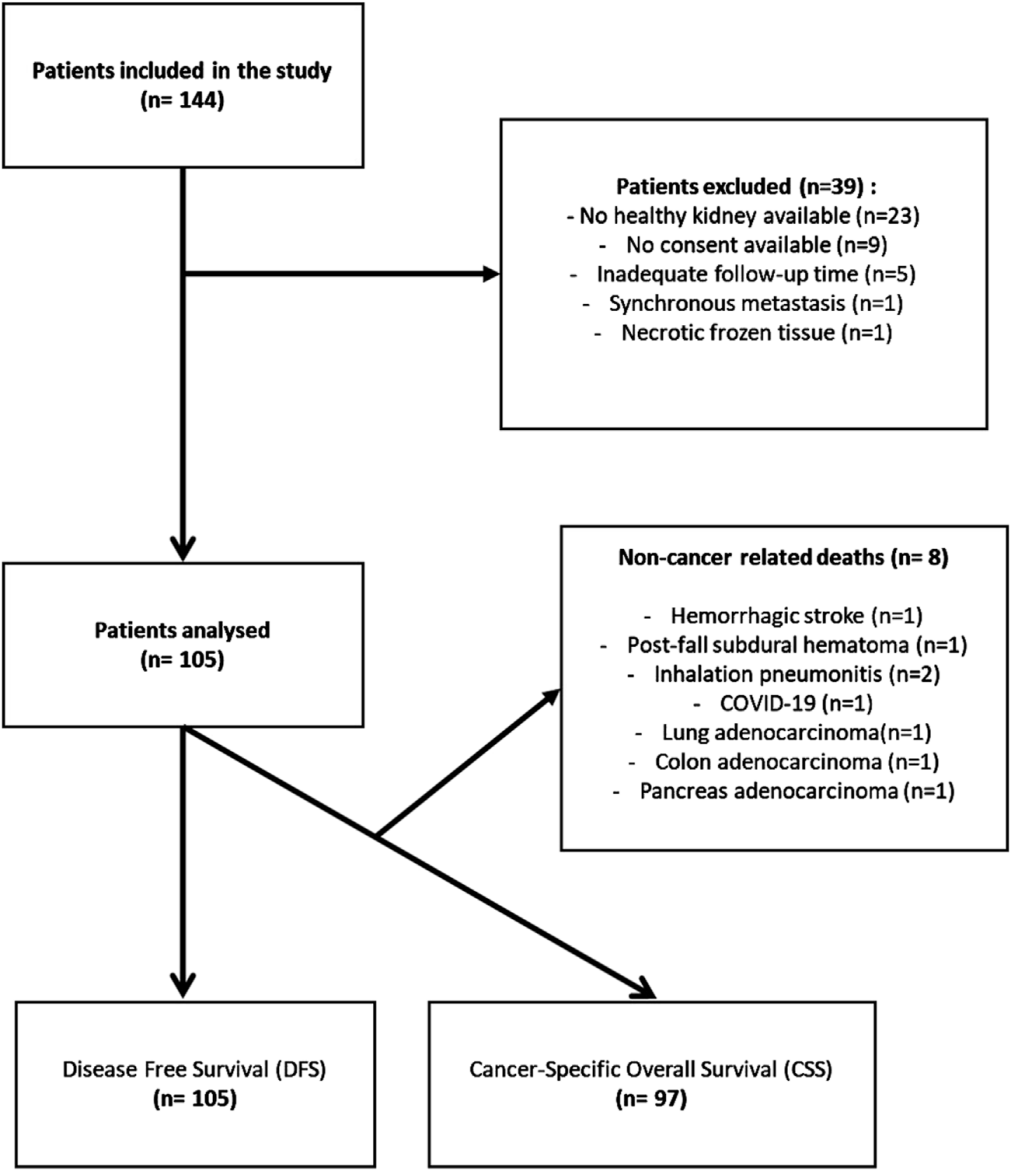
Flow Chart representing the patient’s inclusion process and group analyses.

One hundred and five patients constituted the final cohort of our study. The inclusion process is summarized in Figure 1.

In the overall cohort, the median age was 64 years [56.0–73.0], and the sex ratio (Male/Female) was 1.7:1. The median follow-up was 53 months [36.0–78.0]. Demographic, histologic, clinical and molecular data are summarized in Table 1. Four patients received adjuvant therapy. One patient received Vascular Endothelial Growth Factor (VEGF) inhibitors and an immune checkpoint inhibitor, one patient received VEGF inhibitors only, and two patients received immune checkpoint inhibitors.

**TABLE 1:** Clinicopathological and molecular characteristics of the study population according to HIGH and LOW groups.

	*LOW (n = 53)*	*HIGH (n= 52)*	*p-value*
Age at diagnosis mean (standard deviation)	63.1 (±11.5)	64.6 (±12.5)	0.51
>60 years old n (%)	33 (62%)	33 (63%)	0.89
<60 years old n (%)	20 (38%)	19 (37%)	
Sex			0.59
Men n (%)	32 (60%)	34 (65%)	
Women n (%)	21 (40%)	18 (35%)	
ISUP Grade			0.06
1–2 n (%)	19 (36%)	10 (19%)	
3–4 n (%)	34 (64%)	42 (81%)	
ISUP grade corrected			0.011^a^
1–2 n (%)	48 (91%)	37 (71%)	
3–4 n (%)	5 (9.4%)	15 (29%)	
Tumor Size (standard deviation)	6.17 (±2.60)	6.39 (±2.40)	0.64
pT			0.20
1–2 n (%)	29 (55%)	22 (42%)	
3–4 n (%)	24 (45%)	30 (58%)	
pN			0.31
Nx-N0 n (%)	53 (100%)	51 (98%)	
N1-N2 n (%)	0 (0%)	1 (1.9%)	
Necrosis n (%)	18 (34%)	20 (38%)	0.63
Lymphovascular invasion n (%)	22 (42%)	19 (37%)	0.60
Sarcomatoid or Rhabdoid pattern n (%)	2 (3.8%)	3 (5.8%)	0.63
Positive surgical margin	0 (0%)	1 (1.9%)	0.31
Surgical specimen			0.13
Nephrectomy	41 (77%)	46 (88%)	
Tumorectomy	12 (23%)	6 (12%)	
UISS group, n(%)			0.030*
Intermediate	35 (66%)	38 (73%)	
High	6 (11%)	11 (21%)	
Low	12 (23%)	3 (5.8%)	
SSIGN group, n(%)			0.28
Intermediate	26 (49%)	31 (60%)	
Low	23 (43%)	15 (29%)	
High	4 (7.5%)	6 (12%)	
GRANT group, n(%)			0.17
Favorable	50 (94%)	45 (87%)	
Unfavorable	3 (5.7%)	7 (13%)	
Morphology n(%)			0.005*
Clear	36 (68%)	21 (40%)	
Eosinophilic	17 (32%)	31 (60%)	
Relapse, n(%)	17 (32%)	23 (44%)	0.19
Adjuvant therapy, n(%)	3 (5.7%)	1 (1.9%)	0.31
Disease-Free Survival (months, mean)	45.6 (±29.5)	40.5 (±32.7)	0.49
Specific Overall Survival (months, mean)^a^	62.8 (±34.0)	56.4 (±30.3)	0.44
Ratio mtDNAcn (tumor/healthy kidney), mean (standard deviation)	0.21 (±0.0705)	0.81 (±0.950)	<0.0001*

^a^ Overall survival analysis was performed for 97 patients. ISUP: International Society of Urological Pathology, pT: pathological Tumor status, pN: pathological Node status, UISS: UCLA Integrated Staging System, SSIGN: Stage Size, ISUP Grade, Necrosis, GRANT (Grade, Age, Node, Tumor). *Means significant difference.

### Clinicopathological features and mtDNAcn quantification

In the overall cohort, mtDNAcn was significantly higher in healthy tissue than in tumor tissue, ranging from 52 to 1070 copies (mean:162; [118–256]) in tumor tissue (T) and from 79 to 1920 copies (mean: 569; [368–734]) in the healthy kidney (HK) tissue (p < 0.0001*), leading to a mtDNAcn ratio T/HK ranging from 0.08 to 6.24 [0.22–0.49]. The mean mtDNAcn in healthy tissue was not statistically different in patients who had renal failure (n = 5, *p = 0.21*). The cohort was then divided into two groups, HIGH and LOW, according to the median of this ratio. All the results reported hereafter concern both groups: the HIGH group corresponding to the less mtDNA-depleted tumors and the LOW group for the most mtDNA-depleted ones.

The mean mtDNAcn ratio T/HK was significantly higher in the HIGH group than in the LOW group, respectively 0.81 [0.41–1.23] vs. 0.21 [0.15–0.26] (p < 0.0001*). Both ISUP grade corrected 3-4 and eosinophilic morphology were more frequent in the HIGH group than in the LOW group (p = 0.011* and p = 0.005* respectively). The LOW group comprised more patients of low risk according to the UISS classification (p = 0.030*). No difference was seen in SSIGN or GRANT classification groups or in other classical clinical features (Table 1).

### Increased mtDNAcn in ccRCC patients is associated with worse overall survival

**FIGURE 2:**
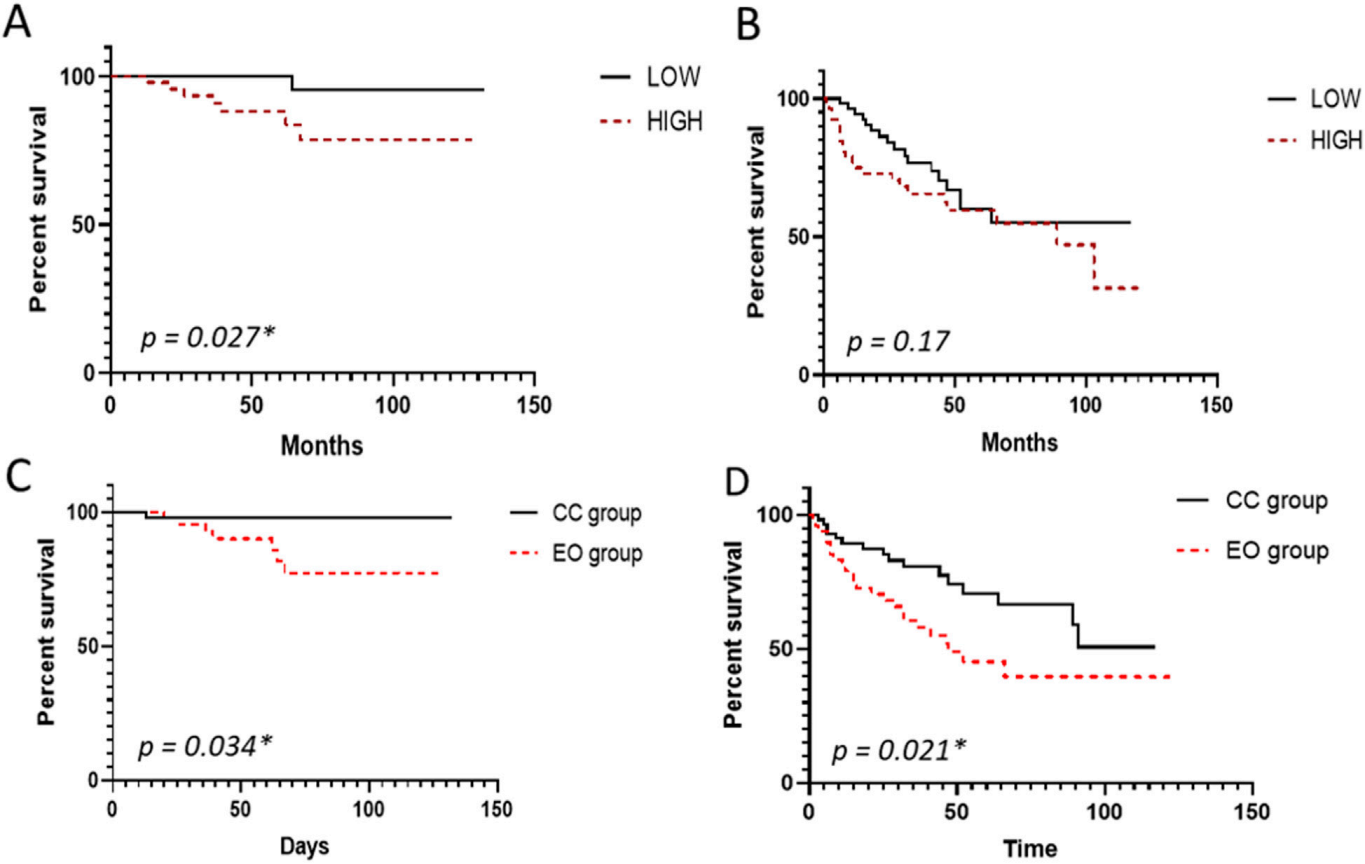
Survival curves according to mtDNA copy number and morphology. Survival Kaplan Meier curve representing **(A)** Cancer-specific survival and **(B)** Disease-free Survival in the HIGH and LOW groups, **(C)** Cancer-specific survival, and **(D)** Disease-free Survival in the Eosinophilic (EO) and Clear Cell (CC) groups.

Cancer Specfic Survival related to ccRCC-death was significantly worse in the HIGH than in the LOW group, with respective 5-year survival rates: 78.7% (CI 95: 64.8–95.5) and 95.5% (CI 95: 87.1–100.0) (Hazard Ratio HR: 7.4 (CI95: 1.9–29.9, p = 0.027*) (Figure 2A). In univariate analysis of CSS, tumor size, ISUPc grade, lymphovascular invasion, and mtDNAcn ratio were significantly associated with a worse prognosis. Only the mtDNAcn ratio was associated with a worse prognosis in multivariate analysis (p = 0.008*) (Table 2). There was no difference in DFS between the two groups (*p = 0.17*) (Figure 2B). Deaths related to ccRCC were significantly more prevalent in the HIGH group than in the LOW group (p = 0.021*, Odds Ratios (OR): 7.8 (CI95: 0.92–66.3). No difference was found regarding relapses (*p = 0.56*).

**TABLE 2:** Univariate and multivariate statistical analyses of specific overall survival by the Cox model.

	Specific overall survival analyses. Cox model; proportional hazards adjustment			
	Univariate analysis		Multivariate analysis	
	HR (CI 95%)	p Value	HR (CI 95%)	p Value
HIGH vs. LOW ratio Groups (mtDNAcnT/HK)	4,79 (1.20; 9.22)	0.020*	13.96 (1.84; 313.94)	0.008*
pT (3–4 vs. 1–2)	7.39 (1.75; 31.32)	0.007*	0.64 (0.03; 14.25)	0.77
pN (1–2 vs. 0-X)	0.36 (1.29e-24–1026)	0.80	3.86e-11 (0; 19.03)	0.29
ISUP grade (3–4 vs. 1–2)	3.59 (0.66; 19.40)	0.13	3.31e+08 (0.30; 1.06e+62)	0.21
ISUPc grade (3–4 vs. 1–2)	6.86 (1.2; 39.30)	0.030*	0.87 (0.16; 5.02)	0.87
Necrosis (presence vs. absence)	2.45 (0.60–10.06)	0.21	1.45 (0.31; 8.21)	0.63
Lymphovascular invasion (presence vs. absence)	4.66 (1.12; 19.42)	0.034*	12.65 (0.98; 501.20)	0.05
Sarcomatoid or rhabdoid pattern (presence vs. absence)	3.10 (0.17; 56.58)	0.44	3.40 (0.05; 111.77)	0.50
Adjuvant therapy (yes/no)	1.65 (0.18; 15,41)	0.86	3.11 (0.05; 95.35)	0.52
Eosinophilic (EO) vs. Clear (CC) Group	6.96 (1.74–27.84)	0.034*	1.18 (0.16–11.52)	0.87

ISUP: International Society of Urological Pathology, pT: pathological Tumor status, pN: pathological Node status. *Means significant difference.

### Eosinophilic features were associated with high mtDNAcn and worse prognosis

Cytoplasmic eosinophilia was rated in all tumors: 54% (n = 57) were classified as CC and 46% (n = 48) as EO. The EO group was significantly associated with the “HIGH” group (p = 0.005*). ISUP grade 3–4, and ISUP grade corrected 3-4 and necrosis were more prevalent in the EO group than in the CC group (p = 0.012*, p < 0.001*, p = 0.022* respectively). The mean tumor size was slightly higher in the EO group than in the CC group (6.8 cm [5–8.5] vs. 5.8 [4–7], p = 0.039*). The mtDNAcn was significantly higher in the EO (mean: 235; [163–343]) than in the CC group (mean: 125 [101–168]) (p < 0.0001*). All clinicopathological data related to the EO vs. the CC group are summarized in Supplementary Table S3.

Deaths due to ccRCC were significantly more prevalent in the EO group than in the CC group, with Odds Ratios (OR) of 8.6 (CI95: 1–72.6; p = 0.021*). No difference was seen in relapses (*p = 0.05*).

In univariate analysis, the 5-year CSS rate in the EO group was significantly reduced compared to the CC group, respectively 77.3% (CI95: [63.2–94.6]) and 98% (CI95: [94.1–100]) (p = 0.034*) (Figure 2C). The DFS was significantly shorter in the EO group than in the CC group, with 5-year survival rates of 39.6% (CI95: [25.5–61.5] for EO compared to 50.7% CI95: [32.4–79.3] for the CC group (p = 0.021*) (Figure 2D). However, it was not an independent prognostic factor in multivariate analysis for overall specific cancer survival (*p = 0.87*).

**FIGURE 3:**
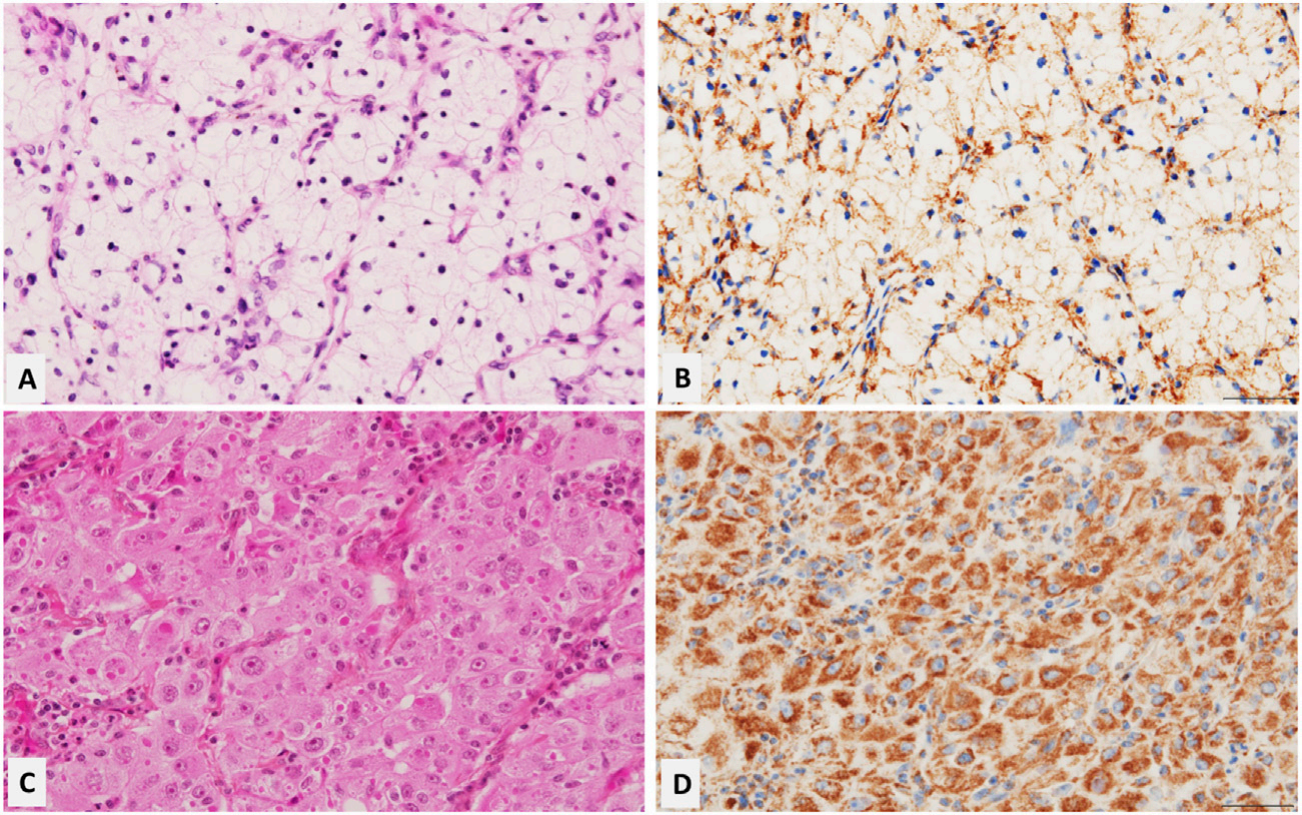
Representative microscopic images of clear and eosinophilic cell features of ccRCC (Hematoxylin, Eosin, Saffron staining, and TOMM20 antibody staining (1/1000) x 100). The clear cell feature of ccRCC shows a weak and submembranous positivity for TOMM20 **(A,B)**. The Eosinophilic feature of ccRCC shows strong and diffuse cytoplasmic positivity for TOMM20 **(C,D)**. This data supports the enrichment of mitochondria in the eosinophilic regions of ccRCC.

Immunohistochemistry showed that TOMM20 was diffusely expressed in EO compared to the CC group (Figure 3), showing that eosinophilic morphology is related to mitochondria and supporting the molecular data showing that EO cells were enriched in mtDNA.

### CcRCC carries mtDNA variants without a mutation hotspot

High-throughput mtDNA NGS was performed on fourteen healthy kidneys and tumor pairs samples. It identified a limited number of somatic mutations in each tumor (1; [1–3.5]). The 39 somatic mutations identified appeared randomly distributed along the mitochondrial genome, regardless of interspecies conservation (Supplementary Table S4). Thirteen mutations were located in non-coding genes (D-loop, ribosomal or transfer RNAs) and 26 in coding genes (Complex I, III, IV, V). No mutational hotspot was identified. Heteroplasmy load was highly variable, ranging from 9.8% to almost homoplasmic variants (88%; [56.75–100]). The number of somatic mutations was not significantly different in the HIGH than in the LOW groups (*p = 0.13*). eKLIPse software [28] did not reveal the accumulation of large-scale mtDNA deletions in tumors (*p = 0.71*) (Supplementary Figure S2).

No statistical difference was found comparing the number of somatic mutations and the EO/CC group (*p = 0.44*). There was no statistical difference in the kind of somatic mutations between CC and EO groups (heteroplasmic variant: *p = 0.61*, non-coding variant: *p = 0.74*). There was no association between mtDNAcn and the number of somatic mutations (*p = 0.21*).

### Depletion of mtDNAcn inhibits the proliferation of 786-0 cells *in vitro*

**FIGURE 4:**
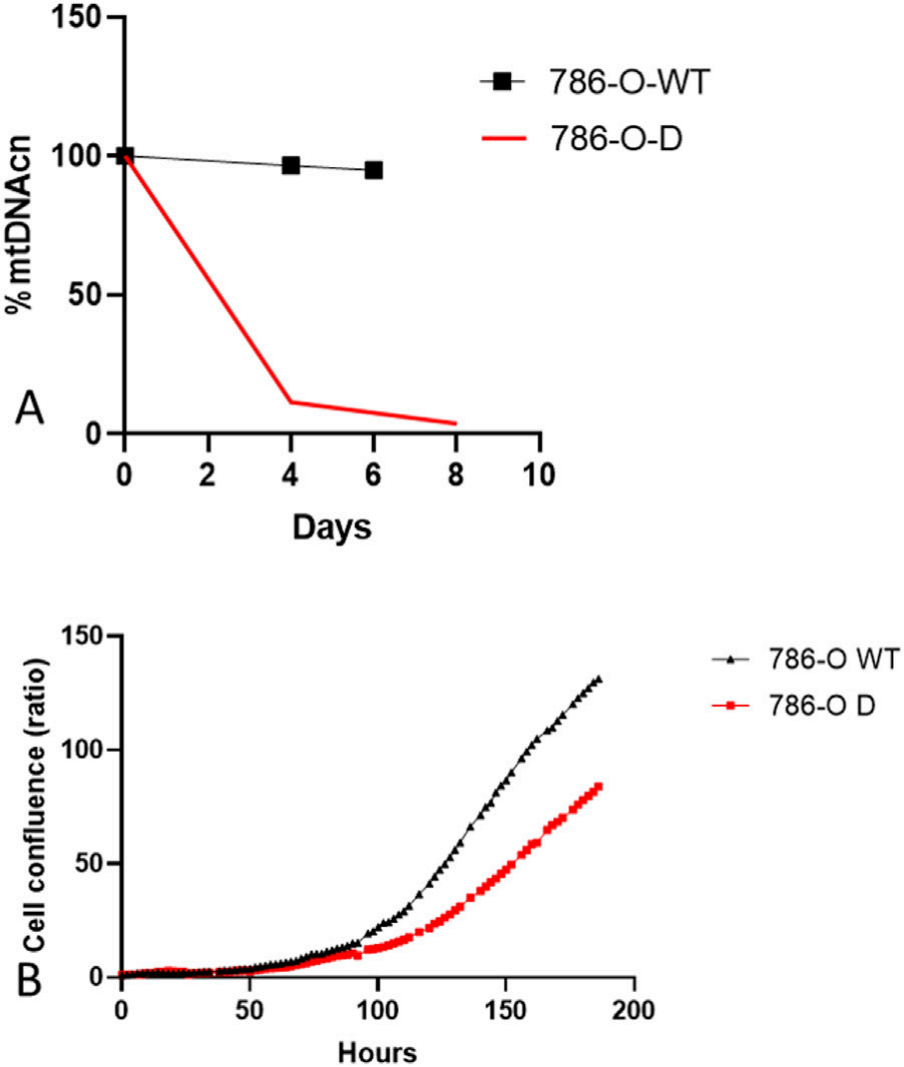
Normalized mtDNAcn and cell proliferation curves according to culture condition. **(A)** mtDNAcn evolution during culture in non-depletion conditions (786-O-WT) and depletion conditions (786-O-D) **(B)** Cell proliferation curves of 786-O-WT cells and 786-O-D were normalised at time 0.

After 4 and 7 days of EB (Ethidium Bromide) exposure, the mtDNAcn of 786-O-D cells was reduced by 88% and 95% compared to untreated 786-O-D cells (Figure 4A). In this *in vitro* assay, mtDNA depletion was associated with a reduction in cell proliferation from day 4. After 7 days of treatment, the 786-O-D growth proliferation rate was 1.8 times slower than untreated cells (p = 0.0005*) (Figure 4B).

## Discussion

Mitochondria play a major role in multiple cellular functions, such as energy production, formation of reactive oxygen species [30, 31], and initiation of apoptosis [30, 32]. Mitochondrial dysfunctions are involved in tumorigenesis [33] through mechanisms of epithelial-mesenchymal transition [34] and metabolic changes (i.e., Warburg Effect) [13–15].

MtDNAcn variations during carcinogenesis are highly dependent on cancer subtypes [22–24, 35]. As previously described [22, 26], we observed that most ccRCCs showed an overall mtDNA depletion compared to the normal kidney. This finding is probably due to the loss of function of the VHL complex secondary to a biallelic inactivation of *VHL* [35, 36], which is present in more than 90% of ccRCCs. Indeed, VHL loss of function induces an abnormal activation of HIF, responsible for ccRCC’s distinctive metabolic reprogramming [36–38], the “Warburg effect” [16, 17], which promotes the shift of glucose use toward the lactate pathway instead of Oxidative Phosphorylation (OXPHOS) [13, 15]. In addition, through the inhibition of carnitine palmitoyltransferase 1A (CPT1A), HIF is responsible for reducing fatty acid transport into the mitochondria and compels the storage of fatty acids as lipid droplets in the cytoplasm [39]. Those mechanisms may partially explain why ccRCCs are naturally depleted in mitochondria, displaying minimal respiratory capacity [40] and harbouring a clear cell appearance [39]. In this context, as tumor cells appear to depend little on mitochondria (OXPHOS) for their metabolism, they may downregulate the level of mtDNAcn.

The effects of mtDNA depletion in *in vitro* models depend on the cell line origin and types of cancer. In breast cancer cell lines, for example, the mtDNAcn reduction slows down the cell proliferation rate and diminishes the tumorigenic phenotype [41, 42]. In contrast, glioblastoma cell lines maintain a tumorigenic phenotype through a low mtDNAcn as a result of a hypermethylation process as previously shown [43]. *In vitro*, we showed that mtDNA depletion decreases cell proliferation in the 786-O cell line, which is derived from a primary renal clear *cell* carcinoma, after being treated with BET. These results support our clinical data, suggesting that a lower mtDNAcn in the tumor is associated with a better prognosis in ccRCC, similar to breast cancer [25]. The opposite data were observed in chromophobe renal cell carcinoma or glioblastoma in young subjects under 40 years old, where an increase of mtDNAcn was associated with better prognosis and survival [22–26]. One limitation is that our *in vitro* study was designed to be exploratory and basic. It could be worthwhile to complete it with an additional essay exploring glucose or glutamine metabolites, for example.

We showed that the tumor mtDNAcn relative to that of healthy tissue was an independent prognostic factor in ccRCC patients. Indeed, the HIGH group ratio was associated with worse overall survival, with a 5-year survival rate of 78.7% compared to 95.5% in the LOW group.

Since 2021, pembrolizumab has exhibited its efficiency in reducing the likelihood of recurrence and mortality following nephrectomy for locally advanced kidney cancer [44, 45]. Despite its positive impact, this treatment induces many side effects, underscoring the importance of identifying patients at the highest risk of recurrence for optimal use in the years ahead. In our cohort, very few patients received adjuvant therapy such as targeted therapy (VEGF inhibitors) or an immune checkpoint inhibitor. The effect of adjuvant treatment did not influence our data. In the same way, there was no statistical difference in mtDNAcn in renal failure patients (n = 5) compared to others, so we believe that despite the morphologic control of frozen healthy renal tissue, it did not cause a bias.

Our results show that, within mitochondrial genetics, mtDNA copy number variation appears to play a more critical role in the physiopathology of ccRCC than the accumulation of somatic mutations. In our cohort, as previously described in renal cancer [11, 35, 46], few mtDNA somatic mutations were identified, mainly carrying low interspecies conservation scores and with a low to moderate impact on mitochondrial function, suggesting a transient rather than a driving role of those variants. However, NGS was performed on a small cohort, which may have limited the detection of certain anomalies due to a lack of power.

CcRCCs are heterogeneous tumors that can display clear or eosinophilic cytoplasm features in the same tumor. As explained above, the clear cell appearance of ccRCC is likely due to multiple mechanisms related to *VHL* inactivation. Moreover, ultrastructural and immunohistochemical studies have shown a higher number of mitochondria in eosinophilic ccRCC than in its clear cell counterpart [47–50]. Interestingly studies of intratumoral heterogeneity showed that the eosinophilic components of ccRCC are associated with poor prognosis outcomes [51, 52] compared to the clear cell components despite a common genetic background with VHL inactivation [18, 53]. In our study, although the diagnosis of ccRCC was done by an experienced pathologist who ruled out differential diagnosis bearing clear cell morphology, we did not sequence the *VHL* gene, which remains a limitation. This limitation is somewhat limited because VHL inactivation is present in almost all ccRCC. Indeed recent advances have identified new entities in renal carcinoma with clear cell morphology and VHL wild type phenotype such as TFE3-rearranged RCC, ELOC (formely TCEB1)-mutated RCC or clear cell renal papillary tumor [54–59].

Our data showed that eosinophilic morphology is associated with increased mtDNAcn and worse DFS and CSS. A recent study [52] has demonstrated the value of distinguishing ccRCCs’ clear cells from eosinophilic cells for the prognostic and therapeutic strategy. Indeed, eosinophilic ccRCCs, due to an abundant lymphocytic inflammatory infiltrate, are good responders to immunotherapy, whereas clear cells ccRCC, characterised by an abundant vascular stroma, have a higher sensitivity to targeted therapy [52]. Nilsson et al. [51] performed RNA sequencing on eosinophilic and clear cell RCC. They showed an overexpression in the eosinophilic component of the DNA polymerase subunit gamma (POLG) encoding for the POLG catalytic subunit, solely responsible for the mtDNA replication. The *POLG* induction may explain the increase of mtDNAcn in this contingent even though we currently do not know the mechanism responsible for *POLG* overexpression. Hence, the accumulation of mtDNAcn in eosinophilic areas is more likely due to an increase in mitochondrial biogenesis rather than a defect in the mitophagy process. Interestingly, in this study, the *TFAM* gene (Transcription Factor A, Mitochondrial) was downregulated, which could be surprising considering its function as an mtDNA transcription factor. However, *TFAM* is also identified as the significant mtDNA packaging protein, constituting the core component of the mitochondrial nucleoid [60, 61]. Hence, an increased *TFAM* expression results in stronger mtDNA compaction, making it less permissive to replication and mitochondrial genome accessibility [62]. As a result, *TFAM* underexpression enables replication consistent with the mtDNAcn increase.

It is important to better understand why and how the increase in mtDNAcn could benefit to ccRCC since it is naturally deprived (see above). One hypothesis is the metabolism of glutamine particularly because this one is favoured by the metabolic acidosis due to the Warburg effect [63, 64]. Glutamine is a non-essential amino acid provided by the diet and synthesised endogenously. Glutamine is further metabolised into glutamate supporting the biosynthesis of nucleotides and amino acids and the energy metabolism throughout the T*ricarboxylic Acid Cycle* (TCA), the OXPHOS via the production of mitochondrial citrate, and fatty acid beta-oxidation [32, 65, 66]. Glutamine would act as a proper energy substrate in this context while the glycolysis and beta-oxidation pathways are reduced [37, 65]. An increase in the number of mitochondria could be associated with an increase in glutaminolysis metabolism, providing the energy and nucleotide substrates necessary for tumor cell proliferation. Evidence supporting this hypothesis relies on the increased isocitrate dehydrogenase *IDH1* gene expression in eosinophilic cells [51]. Hakimi et al [38] reported that tumor progression and metastasis were associated with increased metabolites in the glutathione pathway. Yet, glutathione is a tripeptide made of glutamate, cysteine, and glycine. Moreover, through the formation of glutathione and glutamate, glutamine enables tumor cells to resist to ROS overproduction and anoikis *via* autophagy phenomena [65, 67] and promotes the formation of metastases [65]. Moreover, it has recently been shown that the Solute Carrier Family 1 Member 5 (SLC1A5) transporter [68], which allows the entry of glutamine into the mitochondria, had an oncogenic role since its *in vitro* inactivation was associated with a reduction of tumor growth [68]. Further experiments should be done to investigate the glutamine pathway and metabolites in clear and eosinophilic cells in ccRCC. This hypothesis is supported by several *in vitro* and phase 1-2 studies performed in kidney cancer (papillary or clear cells). These studies showed that glutaminase inhibitors decrease cell growth proliferation and tumor size and potentiate the effects of targeted therapies, especially immunotherapies [69, 70]. Glutamine inhibitors act dually by inhibiting cell proliferation intrinsically (downstream signalling pathways) and by restoring the activation of effector T cells by increasing the availability of cytoplasmic glutamine. As we have seen above, eosinophilic cells are enriched in lymphocytic inflammatory infiltrate and mitochondria, so we would expect immunotherapies combined with glutaminase inhibitors to be beneficial in eosinophilic rather than clear cells ccRCC in future clinical trials.

## Conclusion

In conclusion, high mtDNAcn in tumors compared to healthy kidneys is an independent factor of poor prognosis in ccRCC and is associated with an eosinophilic morphology which highlights the significance of identifying this pattern on pathological examination. The benefit of mtDNAcn increase for the tumor cell could be mediated through the glutamine metabolic pathway. If confirmed, this hypothesis may provide a new perspective on the pathological diagnosis and therapy of ccRCC, especially concerning the synergistic combination of immunotherapy and glutaminase inhibitors.

## Data Availability

The original contributions presented in the study are included in the article/Supplementary Material, further inquiries can be directed to the corresponding author.

## Supplementary material

SUPPLEMENTARY TABLE S1 Clinical, pathological, and demographic data used for statistical analyses. ISUP: International Society of Urological Pathology, pT: pathological Tumor status, pN: pathological Node status, UISS: UCLA Integrated Staging System, SSIGN: Stage Size, ISUP Grade, Necrosis, GRANT (Grade, Age, Node, Tumor).

SUPPLEMENTARY TABLE S2 Primers used for mitochondrial DNA quantitative polymerase chain reaction.

SUPPLEMENTARY TABLE S3 Clinical, pathological, and demographic data of Eosinophilic (EO) vs Clear (CC) groups. ISUP: International Society of Urological Pathology, pT: pathological Tumor status, pN: pathological Node status.

SUPPLEMENTARY TABLE S4 Nature and location of identified mtDNA variants.

SUPPLEMENTARY FIGURE S1 ccRCC’s grading system of eosinophilic features on Hematoxylin-eosin and saffron staining **(**X400): **(A)** 0, **(B)** 1+, **(C)** 2+, **(D)** 3+. **(A)** and **(B)** were merged into the Clear Cell group (CC group) and **(C)** and **(D)** into the eosinophilic group (EO group).

SUPPLEMENTARY FIGURE S2 CIRCOS generated by eKLIPse software representing large scale mtDNA deletions observed in each patient.

## Glossary

786-O-D: 786-O mtDNA depleted
786-O-WT: 786-O wild type
B2M: beta2-microglobulin
*BAP1*: BRCA1-associated protein 1
BCR: Biological Resource Centre
BET: Ethidium Bromide
CC: Clear Cell
CcRCC: Clear Cell Renal Cell Carcinoma
CPT1A: carnitine palmitoyltransferase 1A
CSS: Cancer-Specific Survival
DFS: Disease-Free Survival
DMEM: Dulbecco’s modified Eagle’s medium
EO: Eosinophilic
FBS: Fetal Bovine Serum
FFPE: formalin-fixed and paraffin-embedded
GAPDH: glyceraldehyde 3-phosphate dehydrogenase
HIF: Hypoxia Inducible Factor
IDH1: isocitrate dehydrogenase 1
ISUP: International Society of Urological Pathology
ISUPc: International Society of Urological Pathology Corrected
MtDNA NGS: Mitochondrial DNA Next Generation Sequencing
MtDNA: Mitochondrial genome
MtDNAcn: Mitochondrial DNA copy number
MT-ND1: Mitochondrially Encoded NADH: Ubiquinone Oxidoreductase Core Subunit 1
NGS: Next Generation Sequencing
OS: Overall Survival
OXPHOS: oxidative phosphorylation
*PBRM1*: polybromo-1
POLG: polymerase subunit gamma
Q PCR: Quantitative Polymerase Chain Reaction
ROS: reactive oxygen species
*SCLA1A5*: Solute Carrier Family 1 Member 5
*SETD2*: SET domain containing 2
T/HK: Tumor/Healthy Kidney ratio
TCA: tricarboxylic *acid cycle*
*TFAM*: Transcription Factor A, Mitochondrial
TOMM20: Translocase of the outer mitochondrial membrane complex subunit 20
UISS: University of California Los Angeles Integrated Staging System
UroCCR: French Kidney Cancer Research Network
VEGF: Vascular Endothelial Growth Factor
VHL: Von Hippel Lindau
VLDL-R: low-density lipoprotein receptor
