# Supplementary material for: MtDNA copy number enrichment is associated with poor prognosis and eosinophilic morphology in clear cell renal cell carcinoma
Source: Pathol Oncol Res. 2025 Jul 23;31:1612172. doi: 10.3389/pore.2025.1612172 (PMC12326136; doi:10.3389/pore.2025.1612172)
Supplement: Supplementary file 2 [file Table2.docx]

| Gene | Genome position | Forward primer (5’-3’) | Reverse primer (5’-3’) |
| --- | --- | --- | --- |
| *MT-CO1* | 7017-7036  7205-7224 | TCCACTATGTCCTATCAATA | GGTGTAGCCTGAGAATAG |
| *MT-ND4* | 11297-11306  11501-11520 | CGCACTAATTTACACTCA | GCTAGTCATATTAAGTTGTTG |
| *B2M* | Exon 3 : -112 to -193  Exon 3 : +84 to +113 | CAGCTCTAACATGATAACC | CCTGTAGGATTCTTCTTTC |
| *GAPDH* | Exon 3 : -438 to -421  Exon 3 : -371 to -354 | CCCTGTCCAGTTAATTTC | CACCCTTTAGGGAGAAAA |

**Supplementary Material_Table 2_primers used for mitochondrial DNA quantitative Polymerase Chain Reaction.**
