# Supplementary material for: MtDNA copy number enrichment is associated with poor prognosis and eosinophilic morphology in clear cell renal cell carcinoma
Source: Pathol Oncol Res. 2025 Jul 23;31:1612172. doi: 10.3389/pore.2025.1612172 (PMC12326136; doi:10.3389/pore.2025.1612172)
Supplement: Supplementary file 5 [file Supplementaryfile1.zip › Supplementary Material Figure 2.pptx]

## Slide 1
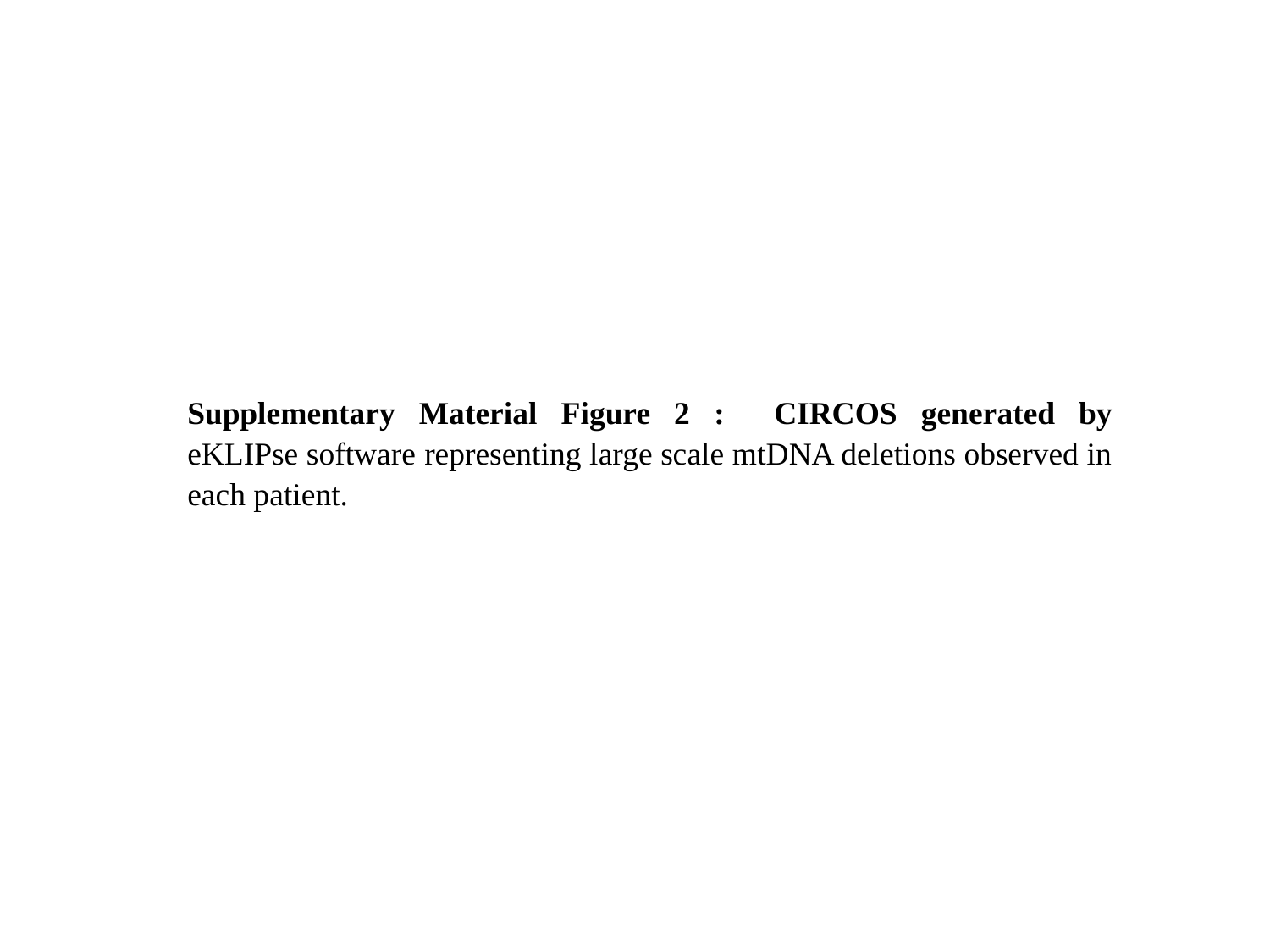

Supplementary Material Figure 2 : 	CIRCOS generated by eKLIPse software representing large scale mtDNA deletions observed in each patient.

## Slide 2
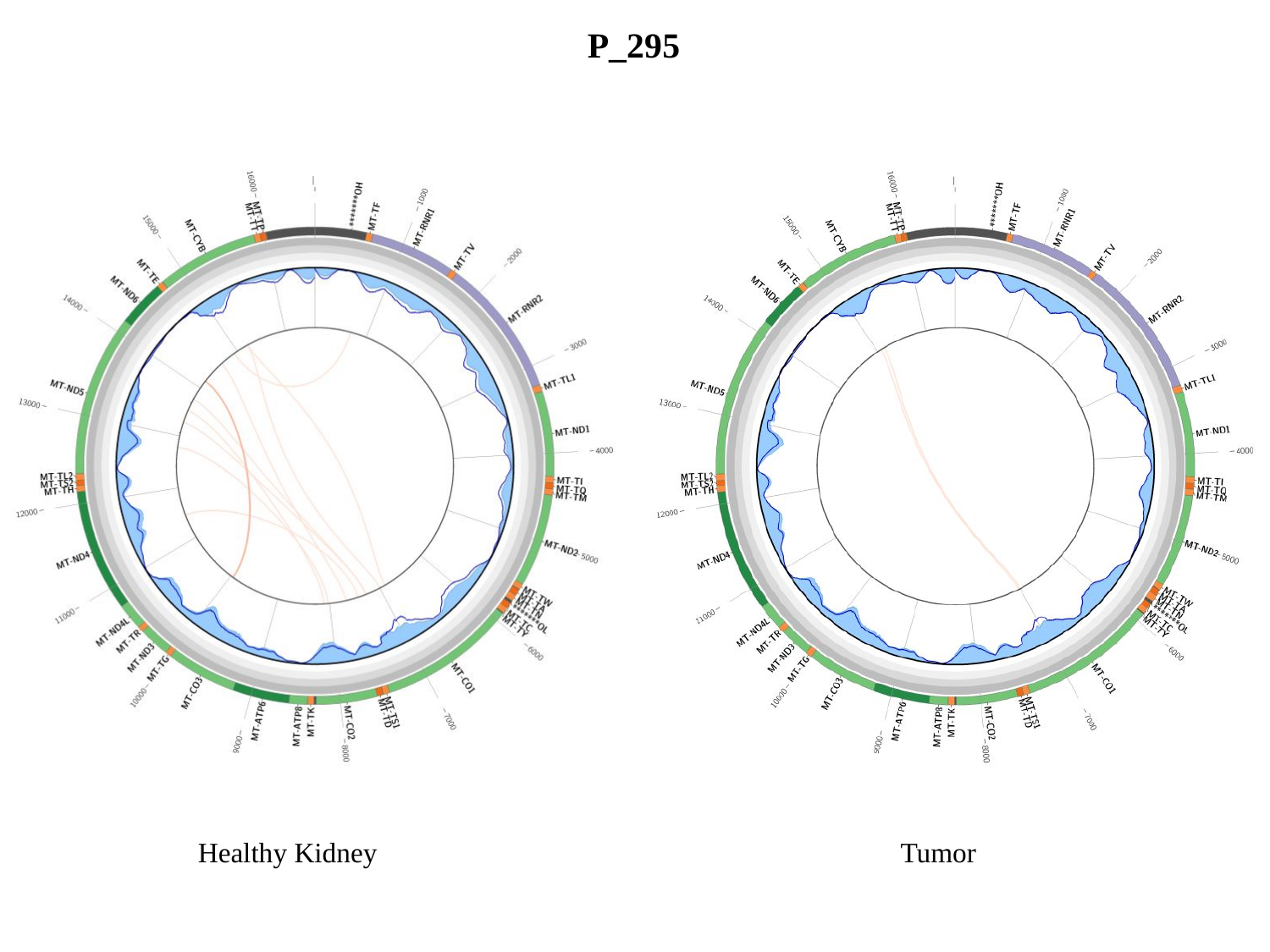

P_295
Healthy Kidney
Tumor

## Slide 3
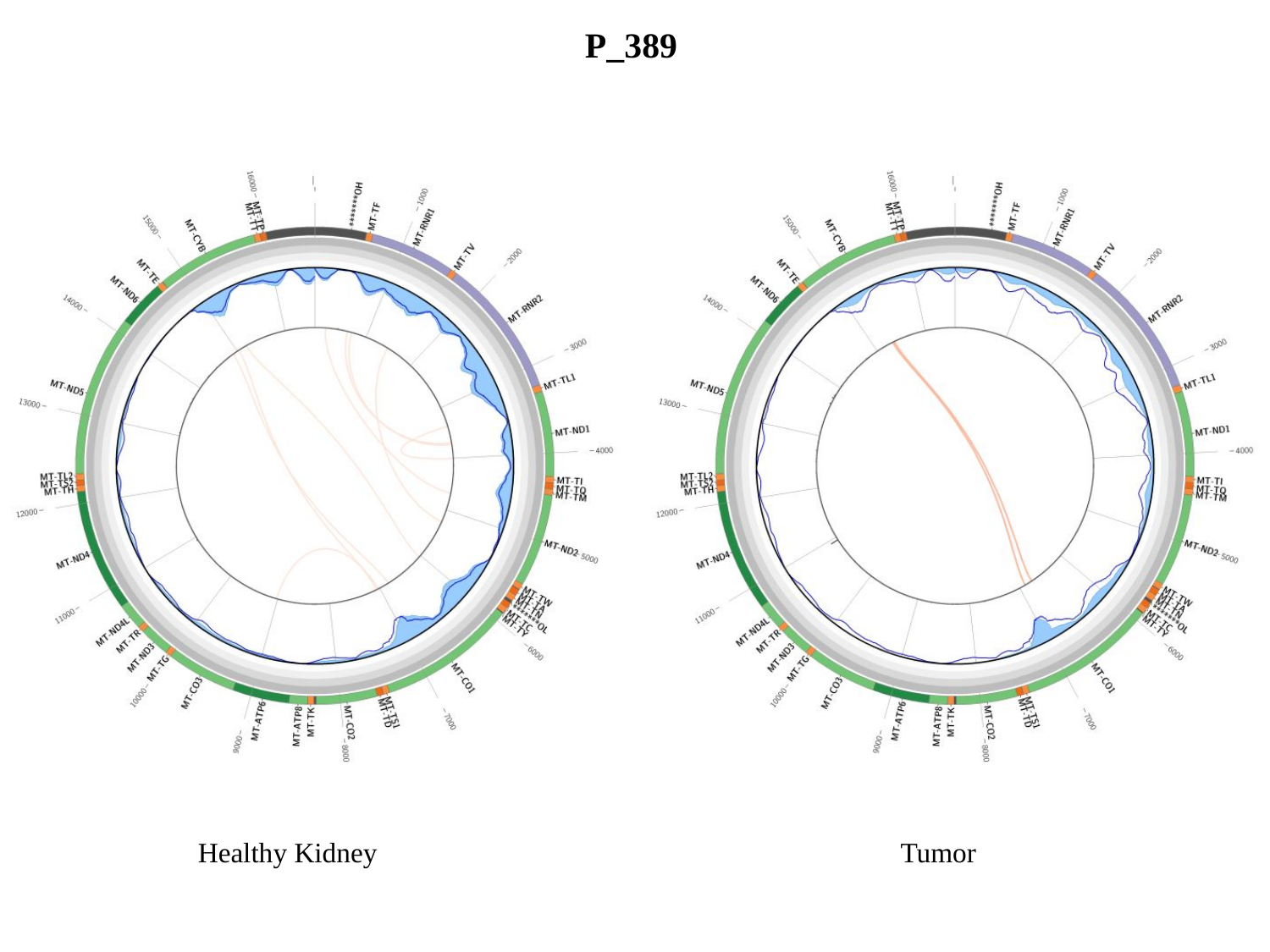

P_389
Healthy Kidney
Tumor

## Slide 4
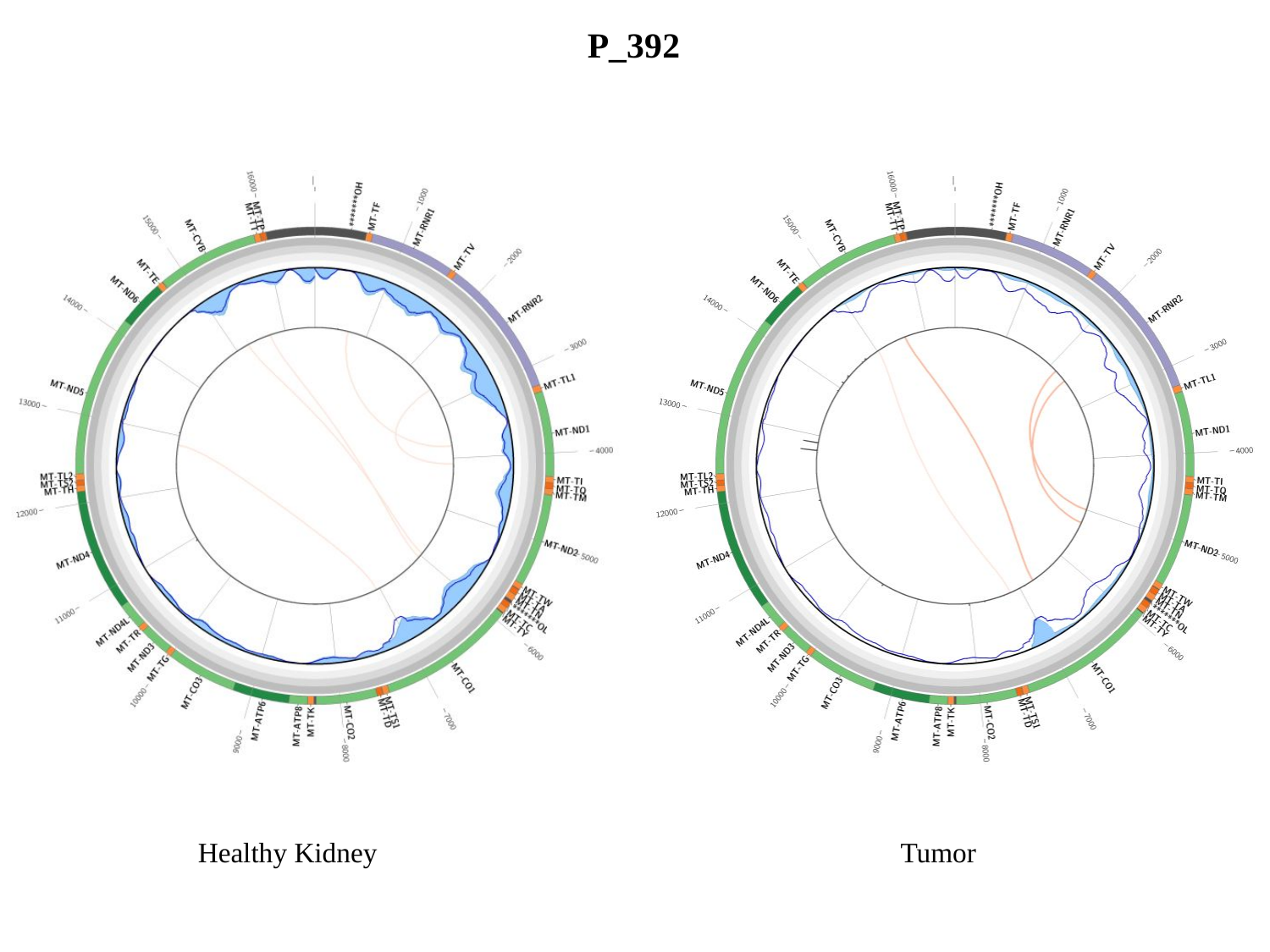

P_392
Healthy Kidney
Tumor

## Slide 5
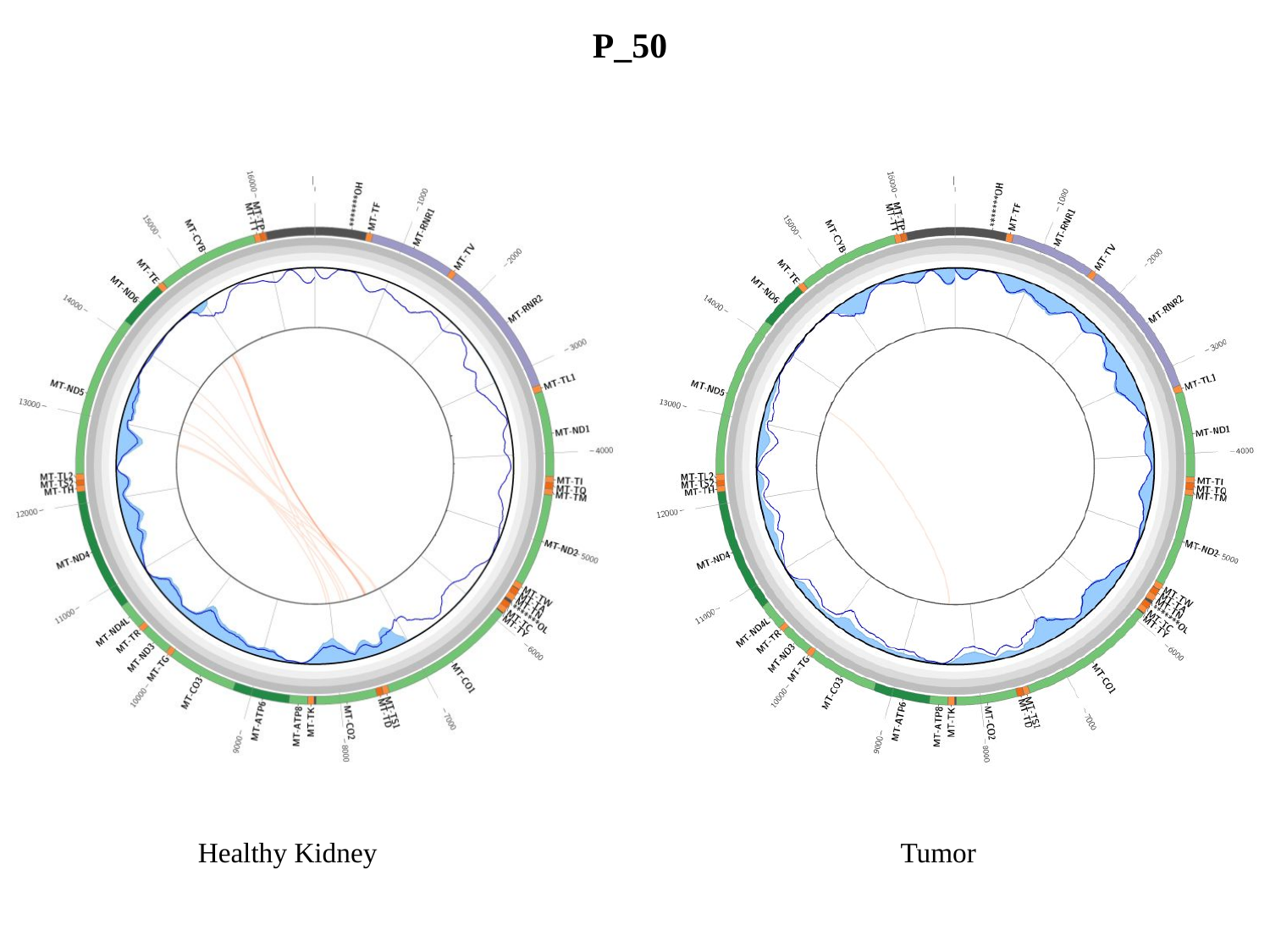

P_50
Healthy Kidney
Tumor

## Slide 6
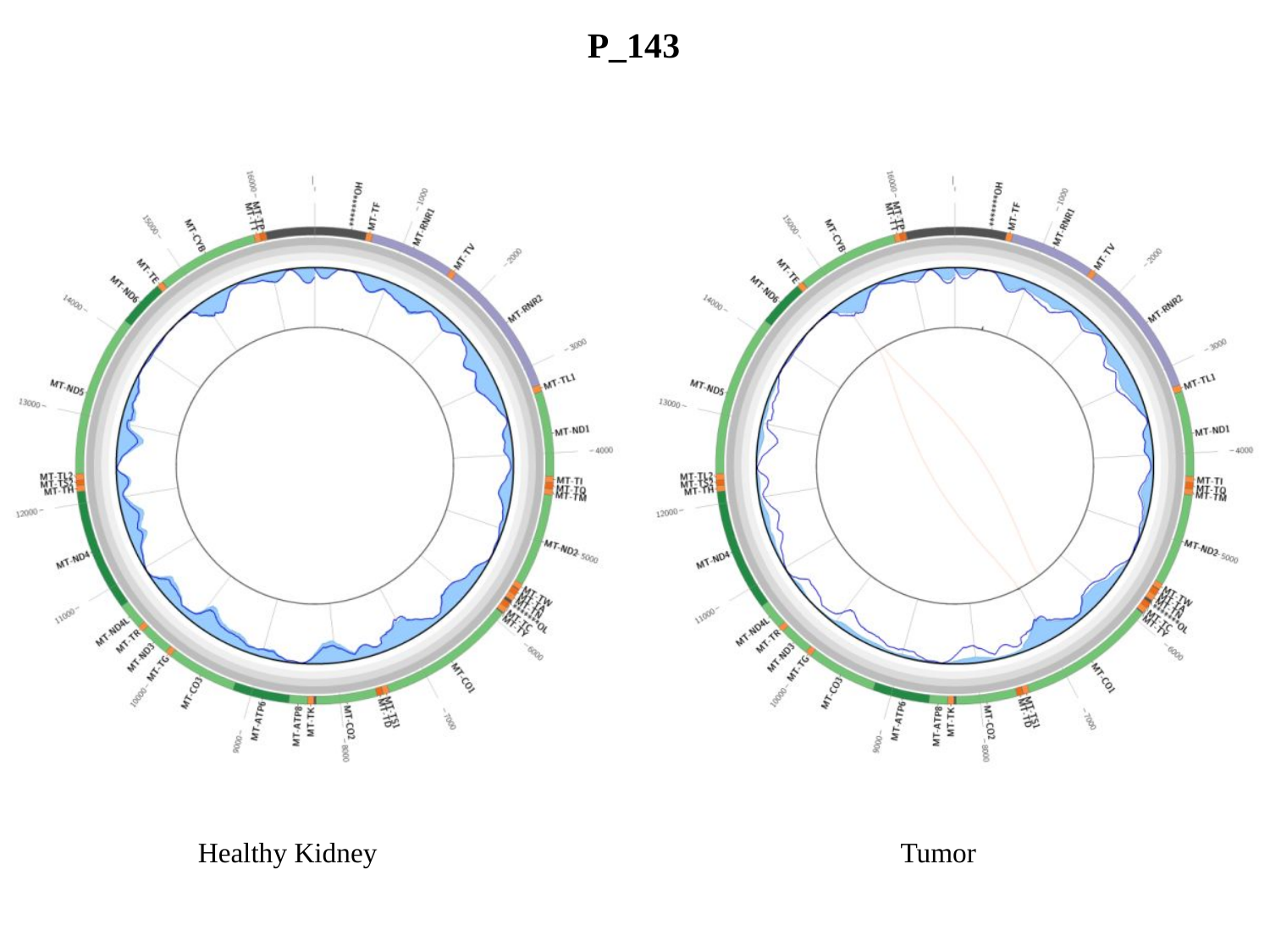

P_143
Healthy Kidney
Tumor

## Slide 7
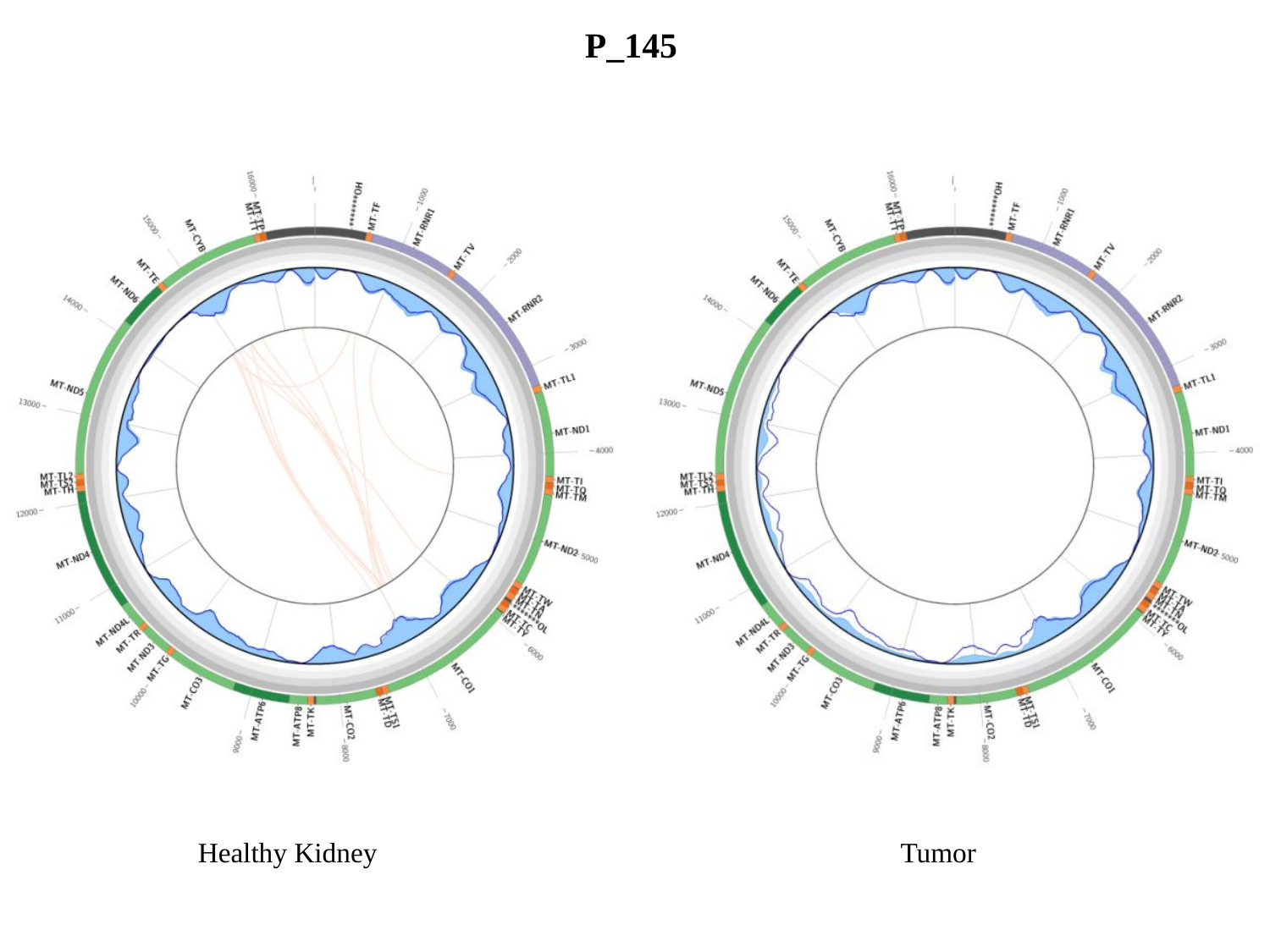

P_145
Healthy Kidney
Tumor

## Slide 8
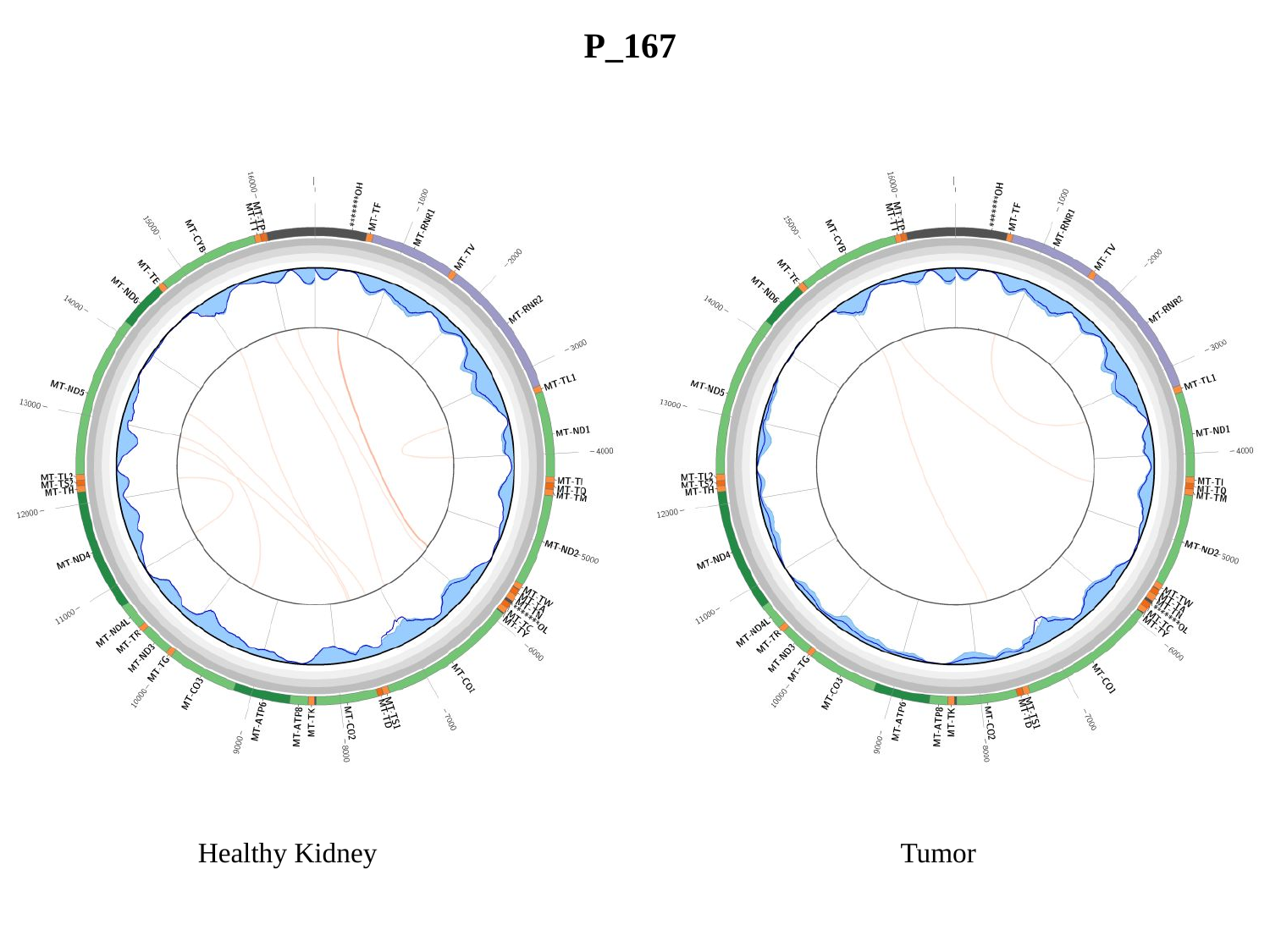

P_167
Healthy Kidney
Tumor

## Slide 9
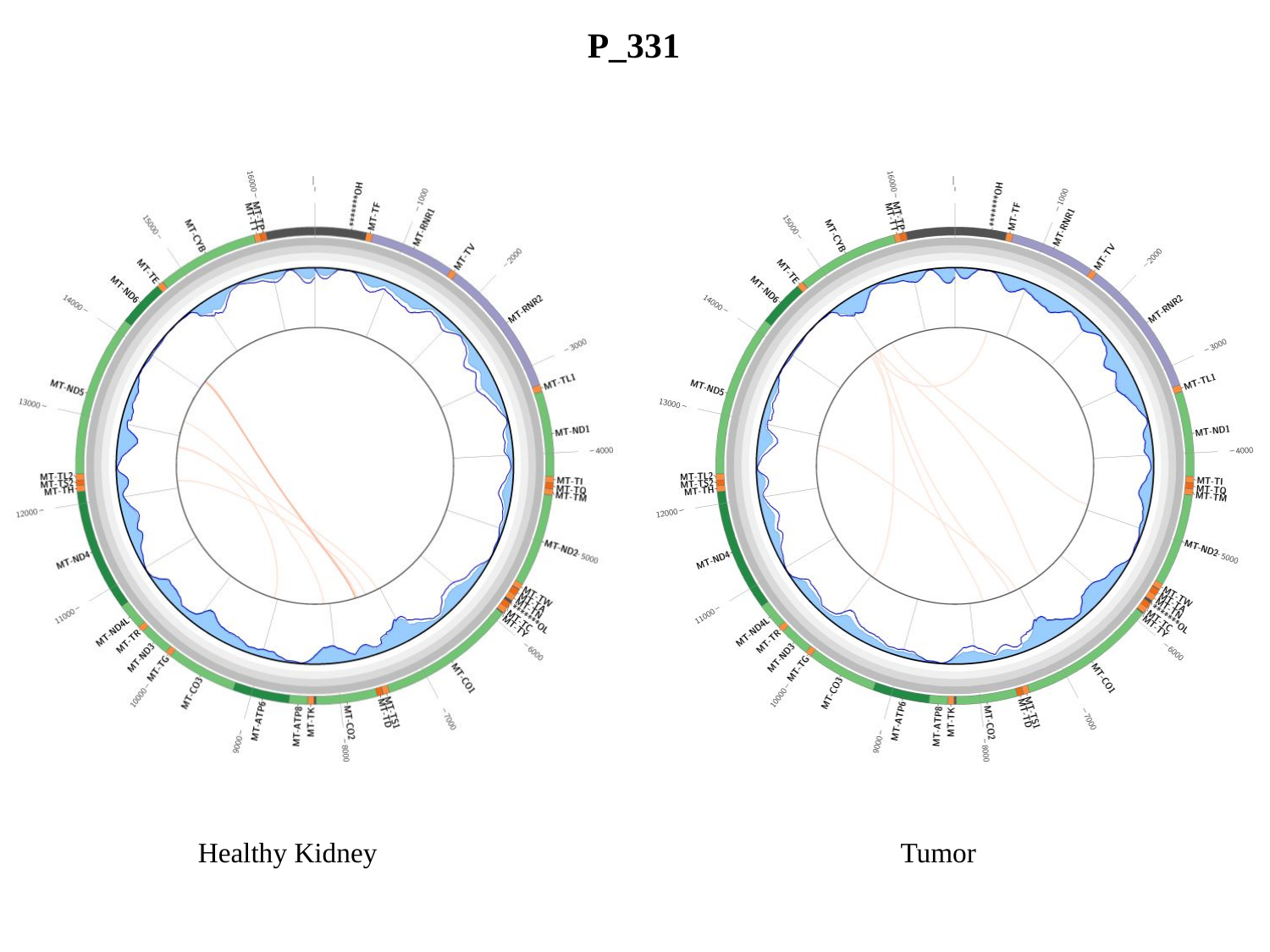

P_331
Healthy Kidney
Tumor

## Slide 10
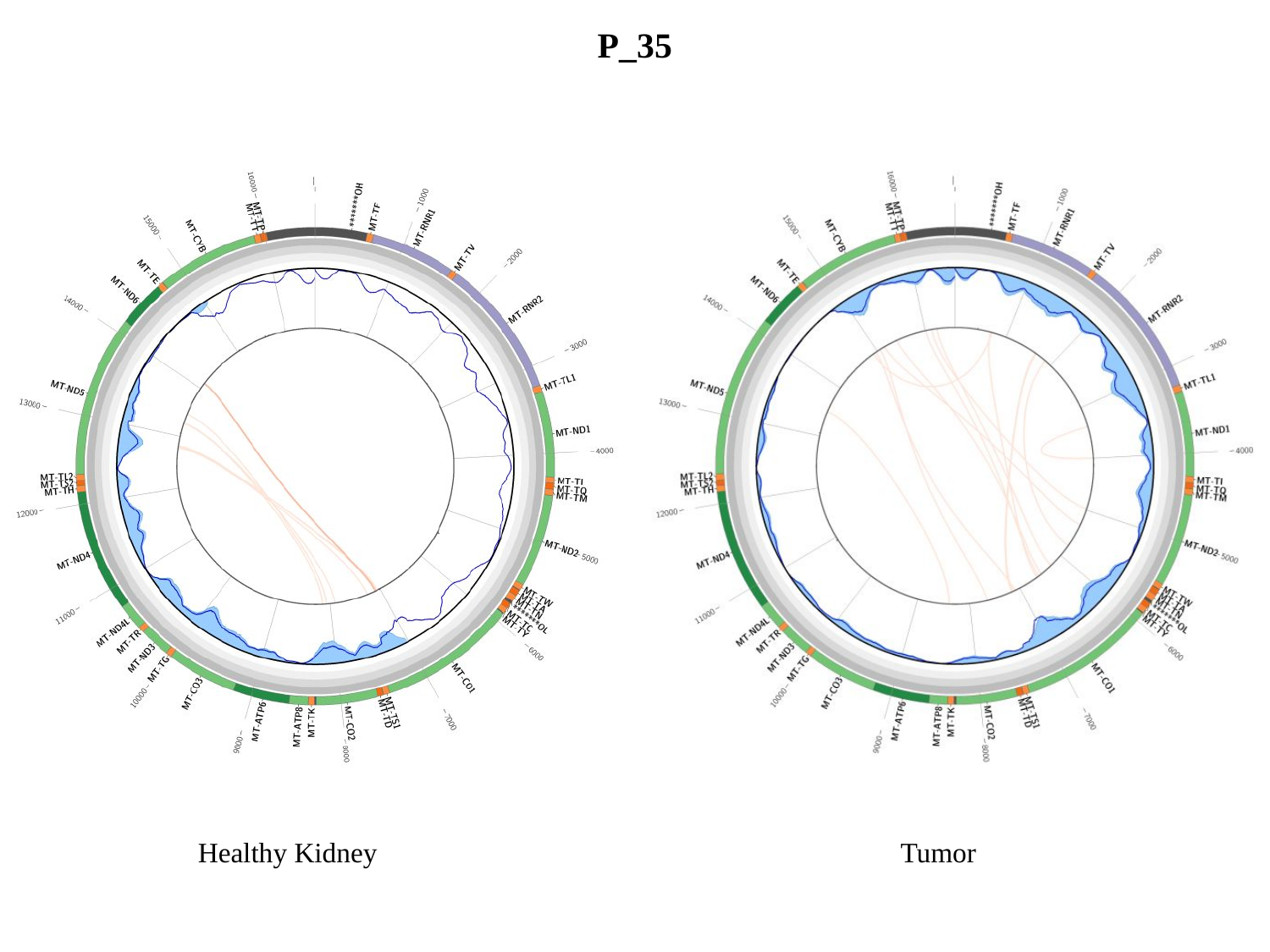

P_35
Healthy Kidney
Tumor

## Slide 11
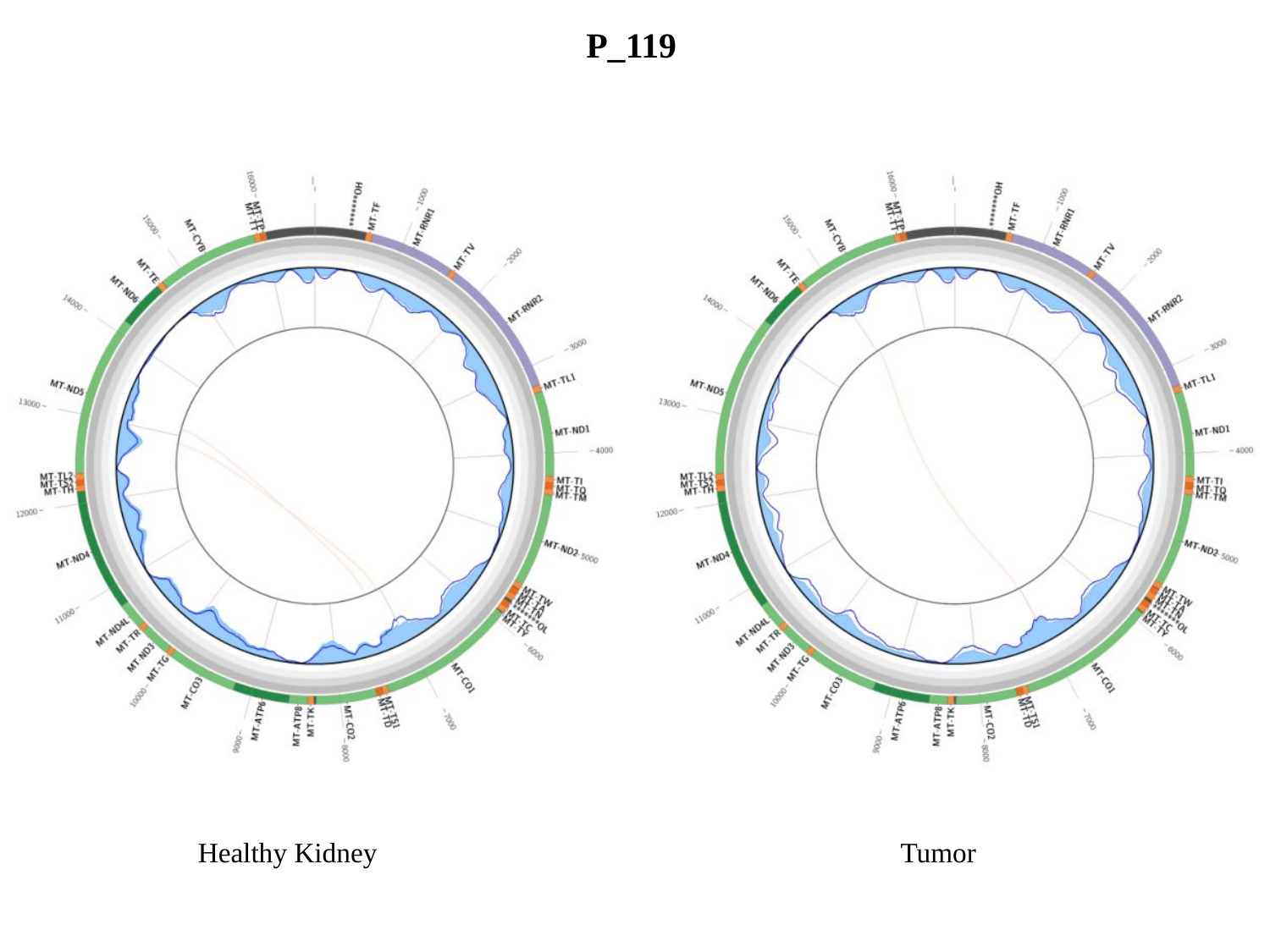

P_119
Healthy Kidney
Tumor

## Slide 12
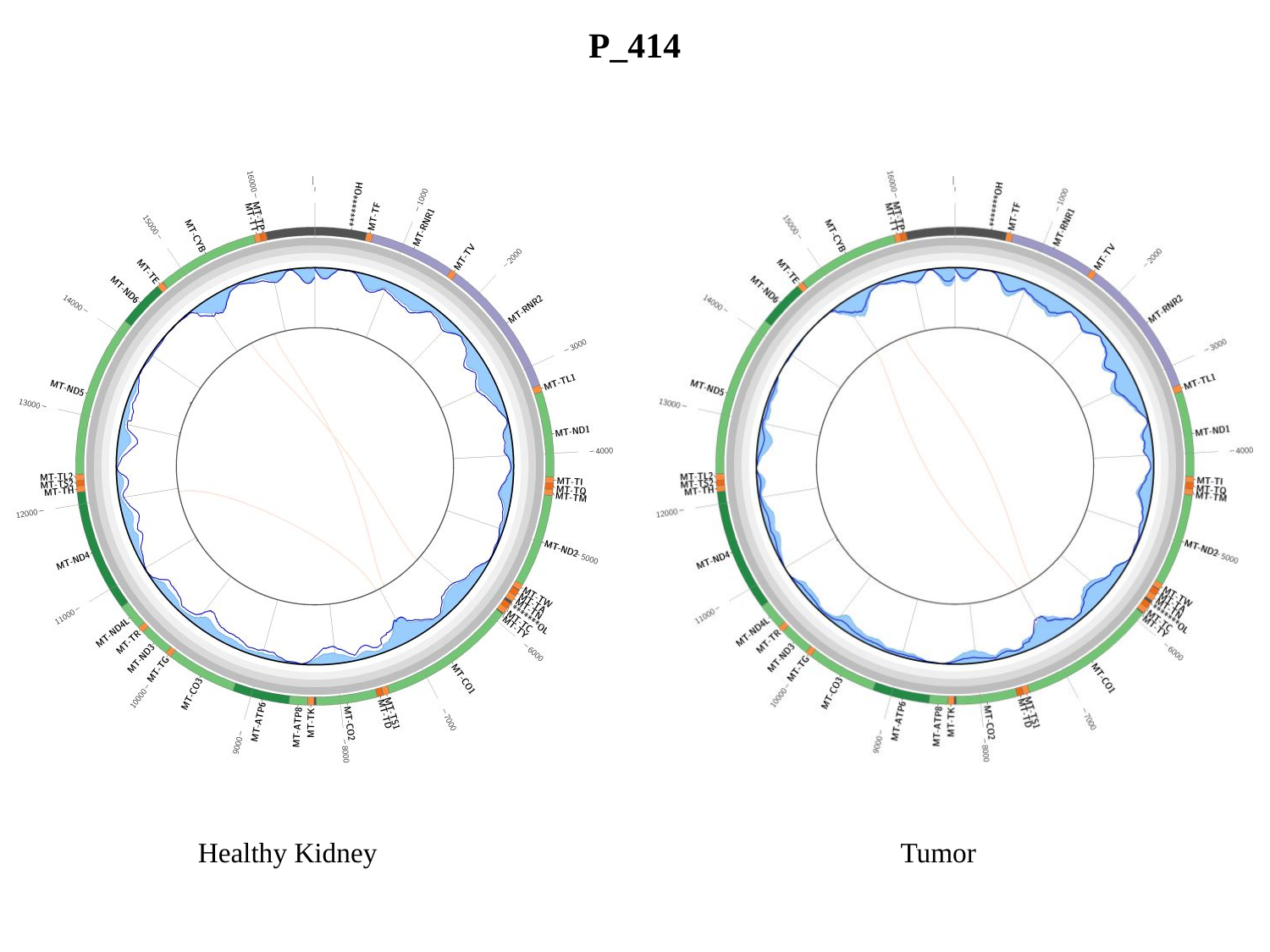

P_414
Healthy Kidney
Tumor

## Slide 13
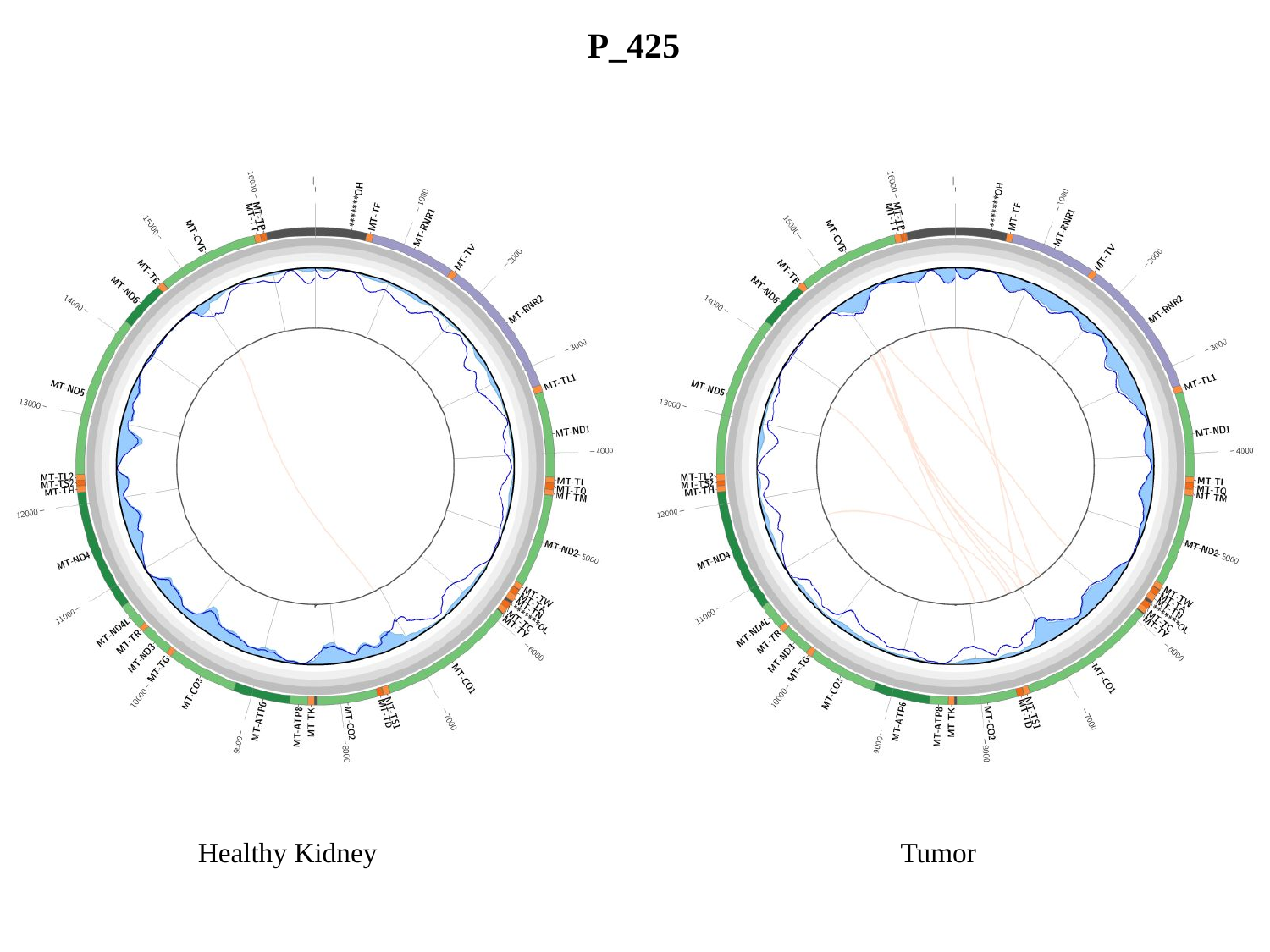

P_425
Healthy Kidney
Tumor

## Slide 14
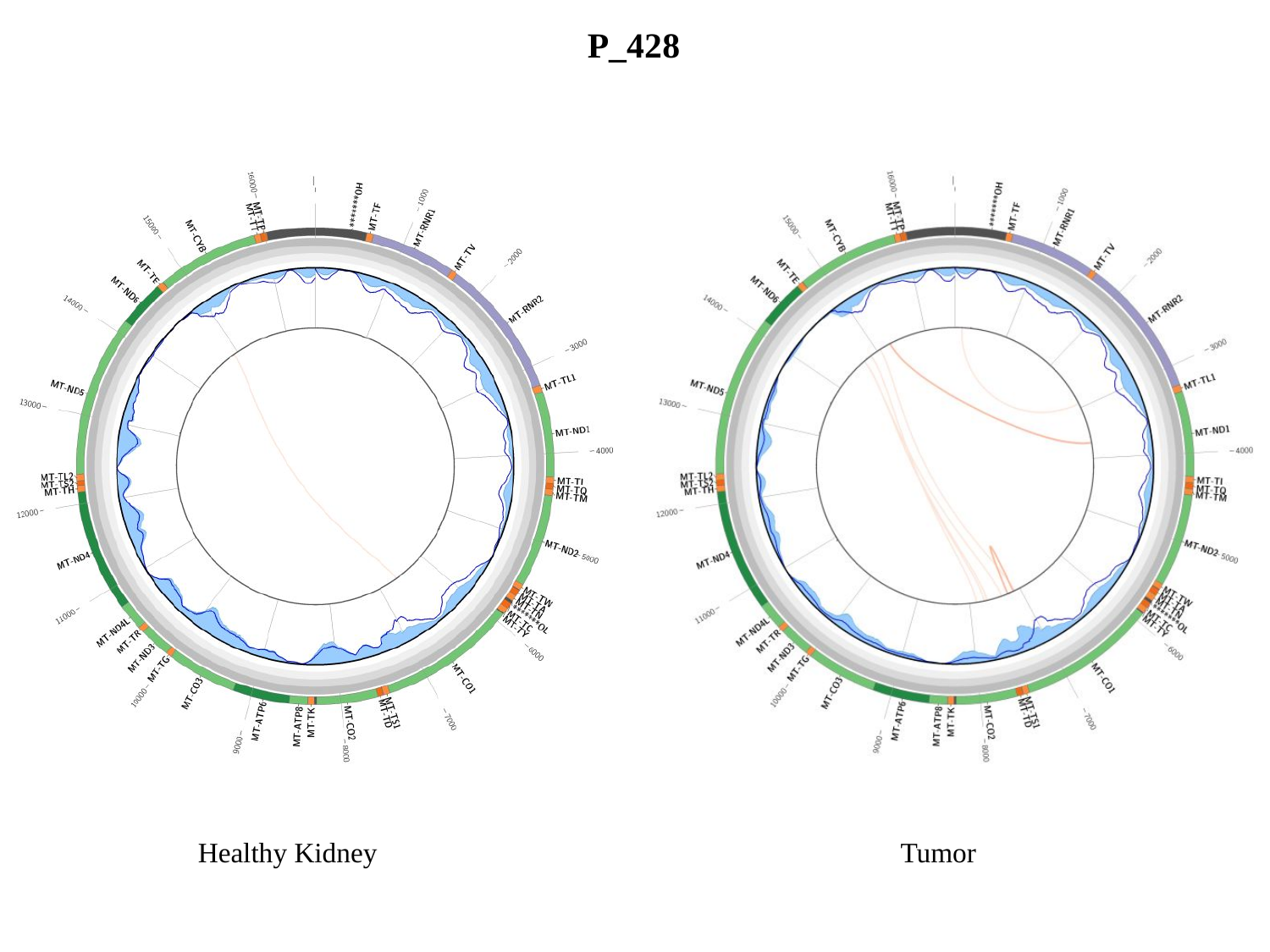

P_428
Healthy Kidney
Tumor

## Slide 15
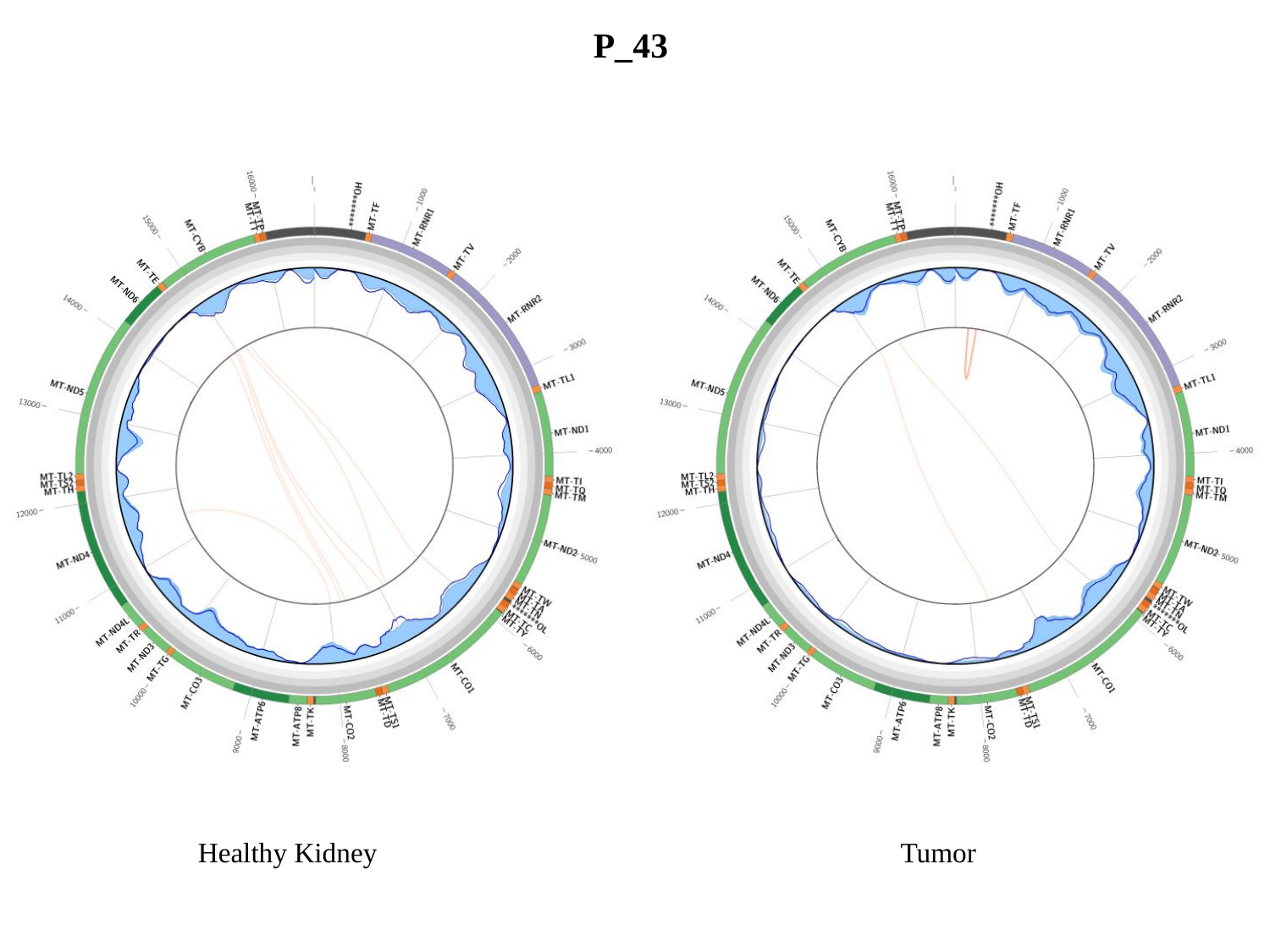

P_43
Healthy Kidney
Tumor
