# Supplementary figures and images for: MtDNA copy number enrichment is associated with poor prognosis and eosinophilic morphology in clear cell renal cell carcinoma
Source: Pathol Oncol Res. 2025 Jul 23;31:1612172. doi: 10.3389/pore.2025.1612172 (PMC12326136; doi:10.3389/pore.2025.1612172)

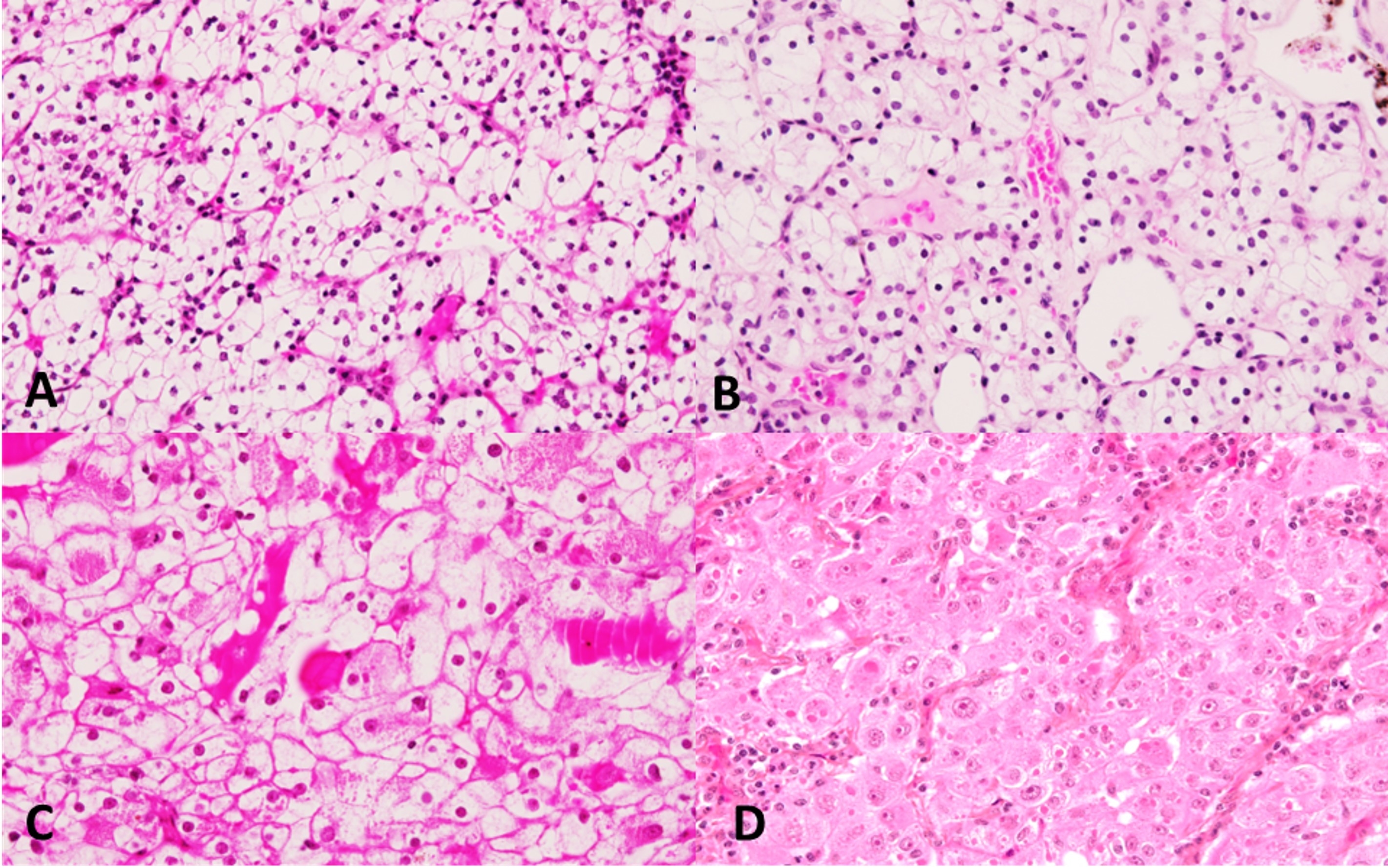

Supplement: Supplementary file 5 [file Supplementaryfile1.zip › Supplementary Material Figure 1.JPEG]
